# Apremilast prevents blistering in human epidermis and stabilizes keratinocyte adhesion in pemphigus

**DOI:** 10.1038/s41467-022-35741-0

**Published:** 2023-01-09

**Authors:** Anna M. Sigmund, Markus Winkler, Sophia Engelmayer, Daniela Kugelmann, Desalegn T. Egu, Letyfee S. Steinert, Michael Fuchs, Matthias Hiermaier, Mariya Y. Radeva, Franziska C. Bayerbach, Elisabeth Butz, Stefan Kotschi, Christoph Hudemann, Michael Hertl, Sunil Yeruva, Enno Schmidt, Amir S. Yazdi, Kamran Ghoreschi, Franziska Vielmuth, Jens Waschke

**Affiliations:** 1 Chair of Vegetative Anatomy, Institute of Anatomy, Faculty of Medicine, LMU Munich, Munich, Germany; 2 Department of Dermatology and Allergology, Philipps-University Marburg, Marburg, Germany; 3 Lübeck Institute of Experimental Dermatology (LIED), University of Lübeck, Lübeck, Germany; 4 Center for Research on Inflammation of the Skin, University of Lübeck, Lübeck, Germany; 5 Department of Dermatology, Rheinisch-Westfälische Technische Hochschule Aachen (RWTH) Aachen University, Aachen, Germany; 6 Department of Dermatology, Eberhard Karl University of Tuebingen, Tuebingen, Germany; 7 Department of Dermatology, Venereology and Allergology, Charité-Universitätsmedizin Berlin, Berlin, Germany

**Keywords:** Skin diseases, Mechanisms of disease, Desmosomes

## Abstract

Pemphigus vulgaris is a life-threatening blistering skin disease caused by autoantibodies destabilizing desmosomal adhesion. Current therapies focus on suppression of autoantibody formation and thus treatments directly stabilizing keratinocyte adhesion would fulfill an unmet medical need. We here demonstrate that apremilast, a phosphodiesterase 4 inhibitor used in psoriasis, prevents skin blistering in pemphigus vulgaris. Apremilast abrogates pemphigus autoantibody-induced loss of keratinocyte cohesion in ex-vivo human epidermis, cultured keratinocytes in vitro and in vivo in mice. In parallel, apremilast inhibits keratin retraction as well as desmosome splitting, induces phosphorylation of plakoglobin at serine 665 and desmoplakin assembly into desmosomal plaques. We established a plakoglobin phospho-deficient mouse model that reveals fragile epidermis with altered organization of keratin filaments and desmosomal cadherins. In keratinocytes derived from these mice, intercellular adhesion is impaired and not rescued by apremilast. These data identify an unreported mechanism of desmosome regulation and propose that apremilast stabilizes keratinocyte adhesion and is protective in pemphigus.

## Abstract (web summary)

Pemphigus vulgaris is a life-threatening blistering skin disease caused by autoantibodies which destabilize cell adhesion of keratinocytes. The phosphodiesterase 4 inhibitor apremilast prevents skin blistering by stabilizing the keratin filament anchorage of desmosomes.

## Introduction

Epidermal integrity is critically dependent on desmosomes, intercellular junctions required for proper adhesion. The adhesive function of desmosomes is maintained by desmosomal cadherins of the desmoglein (Dsg) and desmocollin (Dsc) subfamilies, which are linked to the keratin filament cytoskeleton via several plaque proteins, including plakoglobin (Pg), plakophilins (Pkp) and desmoplakin (Dp) in the epidermis^1,2^. Additionally, desmosomes regulate signaling pathways to adapt cellular behavior and differentiation^3,4^.

Pemphigus is a life-threatening bullous autoimmune disease in which autoantibodies against Dsg1, Dsg3, Dsc3 as well as other antigens cause flaccid blistering in the epidermis and in mucous membranes of the oral cavity and elsewhere^5–7^. Mechanisms leading to loss of keratinocyte cohesion comprise direct inhibition of Dsg3 interaction and dysregulation of a plethora of intracellular signaling pathways upon autoantibody binding^8–11^. Dsg1 and Dsg3 act as signal transducers and regulate autoantibody-specific signaling pathways^12,13^. Signaling molecules contribute to the loss of intercellular adhesion and comprise p38MAPK, PLC, Erk, ADAM10 and Src. In contrast, cAMP represents a cellular rescue mechanism^14–20^. Pemphigus autoantibodies slightly increase cAMP in keratinocytes^20^. This mechanism is sufficient to prevent loss of intercellular adhesion when augmented pharmacologically^20^. Besides, cAMP strengthens cadherin-mediated adhesion in different tissues and enhances desmosomal adhesion in cardiomyocytes via phosphorylation of Pg at S665^21,22^.

In pemphigus, therapeutic strategies range from unspecific immunosuppression, targeted immunotherapy such as immune-apheresis and rituximab depleting autoantibody-producing B cells to experimental methods which are not yet clinically approved^6,23–29^. All these strategies modulate the immune system of the patients and thus are associated with considerable side effects. A precise understanding of the mechanisms leading to loss of intercellular adhesion as well as therapeutic approaches to stabilize desmosomes in keratinocytes would fulfill an unmet medical need.

During the past years, several phosphodiesterase 4 inhibitors were developed and clinically applied^30,31^. Among them, apremilast is clinically approved for treatment of psoriasis and Behcet disease^32–35^. Recently, a pemphigus patient has been treated successfully with apremilast^36^. However, therapeutic effects of apremilast were mainly attributed to the immune system and effects on keratinocytes have not been investigated yet. Here, we demonstrate that apremilast is effective to abrogate epidermal blistering in pemphigus and enhances desmosomal anchorage to the cytoskeleton and thus may represent a new treatment approach in pemphigus.

## Results

### Apremilast is protective in human skin ex vivo and in the pemphigus passive-transfer model in vivo

**Fig. 1:**
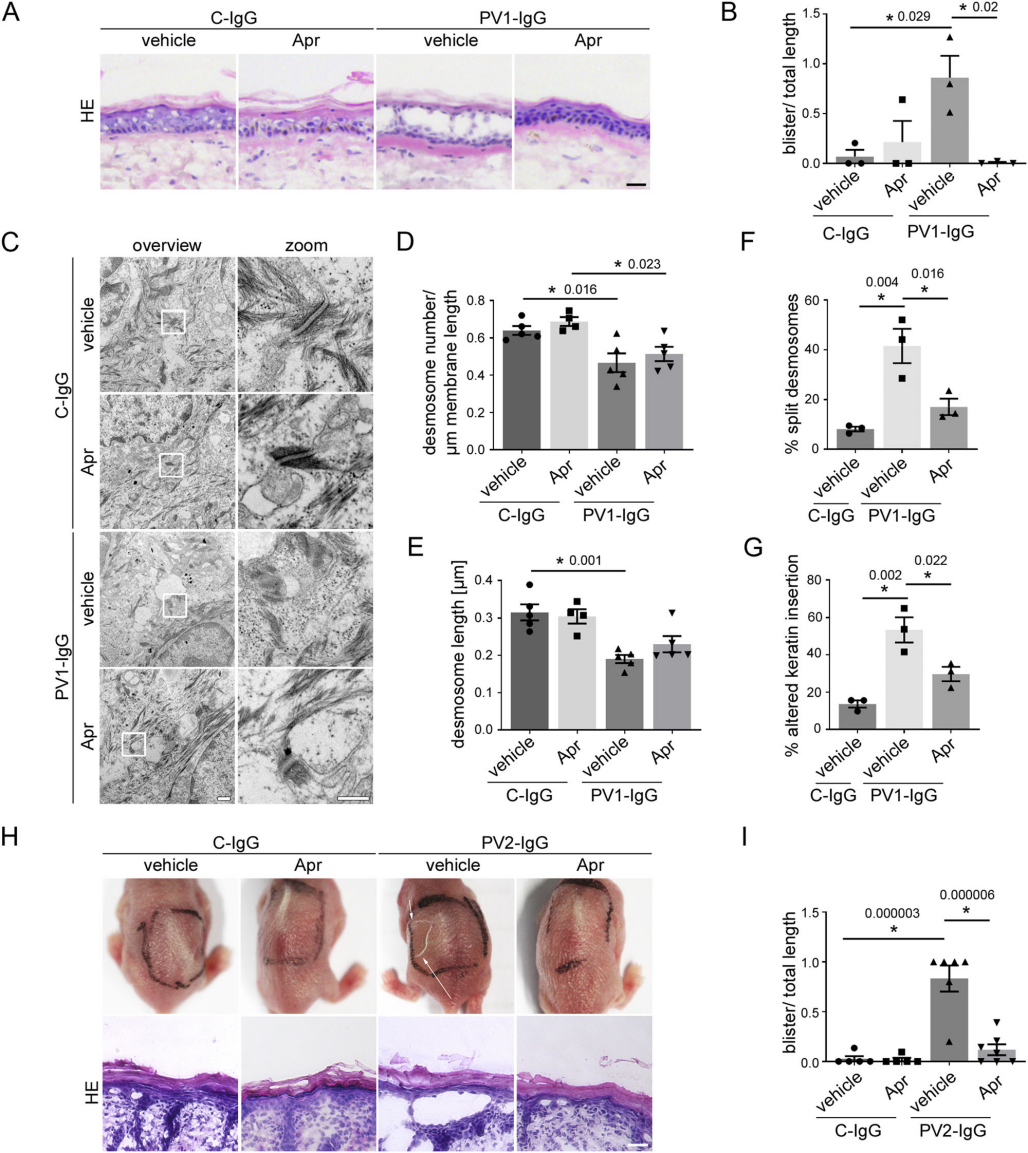
Apremilast is protective in an ex vivo pemphigus skin model and in vivo in a neonatal pemphigus mouse model. **A** HE staining of human ex vivo skin samples from healthy humans after injection of vehicle (DMSO) or apremilast (Apr) prior to injection of PV1-IgG or control (C)-IgG. Apremilast (100 µM) prevented PV1-IgG-induced intraepidermal blister formation. Scale bar = 25 µm. Representative of *n* = 3. **B** Quantification of blister length revealed a significant decrease in blister length after apremilast treatment (*n* = 3). **C** Electron microscopic analysis of desmosome ultrastructure of ex vivo skin samples. Scale bar (overview) = 500 nm. Zoom in areas marked with white rectangles. Scale bar (zoom) = 200 nm. Representative of *n* = 5. Quantification revealed no rescue of PV1-IgG-induced reduction in number (**D**, *n* = 5) and length (**E**, *n* = 5) of desmosomes but showed improvement of PV1-IgG-induced formation of split desmosomes (**F**, *n* = 3) and altered keratin network insertion (**G**, *n* = 3) by apremilast. **H** Macroscopic and microscopic phenotype of epidermis of neonatal pemphigus mouse model. c-IgG-injected samples revealed a normal appearance of the epidermis. PV2-IgG induced suprabasal blistering which was abrogated by apremilast (1 µM) treatment. Scale bar = 25 µm. Representative of *n* > 5. **I** Quantification of blistered epidermis. Apremilast diminished PV-IgG-induced suprabasal epidermal blister formation. (C-IgG samples: *n* = 5; PV-IgG+vehicle: *n* = 6, PV-IgG+Apr: *n* = 7). Columns indicate mean value ± SEM, *P < 0.05 (exact values are depicted in the figure). One-way ANOVA with Bonferroni correction. pemphigus vulgaris (PV). Source data are provided as a Source Data file.

For the human ex vivo skin model, healthy human skin samples from body donors were injected subepidermally with apremilast (Apr) or vehicle (DMSO) followed by either control IgG (C-IgG) or PV1-IgG injection 1 h later. Apremilast was used at a concentration of 100 µM for the ex vivo model, which significantly elevated cAMP levels (Figure S1A, B) to ensure reliable results for quantitative transmission electron microscopy (TEM) analysis. Samples were incubated for 24 h and subsequently subjected to mechanical stress. HE staining of C-IgG-injected samples with and without apremilast showed no blister formation or morphological alteration in the epidermis (Fig. 1A, B). In contrast, PV1-IgG (Table 1) injection induced suprabasal blistering with a mean cleft length of 860 µm (+/−220 µm), which was completely abrogated by injection of apremilast indicating that apremilast prevents blister formation independent of effects on immune cells and thus, most likely, by ameliorating loss of intercellular adhesion in keratinocytes (Fig. 1A, B).

**Table 1:** Dsg autoantibody levels of patient-IgGs

name	Dsg1 [U/ml]	Dsg3 [U/ml]
PV1-IgG	760	4711
PV2-IgG	1207	3906

Levels were analyzed by ELISA of purified IgG samples of pemphigus patients (IgG).

Next, we stained the ex vivo samples for PV antigens Dsg1 and Dsg3. The epidermis showed normal inverse expression pattern of Dsg1 and Dsg3 in C-IgG-treated samples, which resembled Dsg distribution of fresh skin biopsies (Fig. S1C-E)^37–40^. In contrast, PV1-IgG injection led to a fragmented and reduced Dsg1 and Dsg3 staining in the epidermis, further referred to as Dsg depletion as described before^41^. Surprisingly, apremilast did not attenuate PV1-IgG-induced Dsg depletion. The specificity of staining was shown by secondary antibody control (Fig. S1F).

We analyzed desmosome ultrastructure of ex vivo samples using TEM to investigate the protective mechanisms underlying the apremilast effect. Studies on PV patients’ skin biopsies or PV mouse and human ex vivo models revealed an ultrastructural PV-phenotype characterized by reduction of desmosome number and size, uncoupling of keratins from the desmosomal plaque and desmosomes with separated plaques, referred to as split desmosomes^39,42–45^. PV1-IgG-injection reduced desmosome number and size, which was not rescued by apremilast injection (Fig. 1C–E). In contrast, apremilast treatment reduced PV1-IgG-induced formation of split desmosomes (Fig. 1C, F), suggesting that apremilast strengthened adhesion of remaining desmosomes. Keratin uncoupling is a morphological hallmark in pemphigus contributing to destabilization of desmosomes in PV. Vice versa, blocking of keratin retraction is protective against PV-IgG-induced loss of intercellular adhesion^14,45–48^. Thus, we analyzed the insertion of keratin filaments to the desmosome, which was altered upon autoantibody injection (Fig. 1C, G). Importantly, apremilast significantly ameliorated PV-IgG-induced uncoupling of keratins from the desmosome. Taken together, these data suggest that the protective effect of apremilast in PV may be based on improved keratin insertion.

Next, we applied apremilast in the neonatal passive-transfer mouse model in vivo. Because a dose of 1 µM resembles plasma concentration at a common oral administration of 2 × 30 mg/day, this concentration was used for the in vivo murine mouse model (https://www.ema.europa.eu/en/documents/product-information/otezla-epar-product-information_de.pdf).

Neonatal mice were injected subcutaneously with vehicle or apremilast and subsequently received an additional injection with either C-IgG or PV2-IgG 2 h later. After 10 h animals were sacrificed and a defined mechanical stress was applied. C-IgG-treated samples revealed an intact epidermis whereas PV-IgG induced macroscopic blisters (Fig. 1H, upper panel) with a typical suprabasal cleft formation in a HE staining (Fig. 1H, lower panel). Importantly, apremilast at 1 µM was sufficient to ameliorate PV-IgG-induced acantholysis (Fig. 1H, I).

### Apremilast abrogates PV-IgG induced loss of cell adhesion and keratin alterations in vitro

**Fig. 2:**
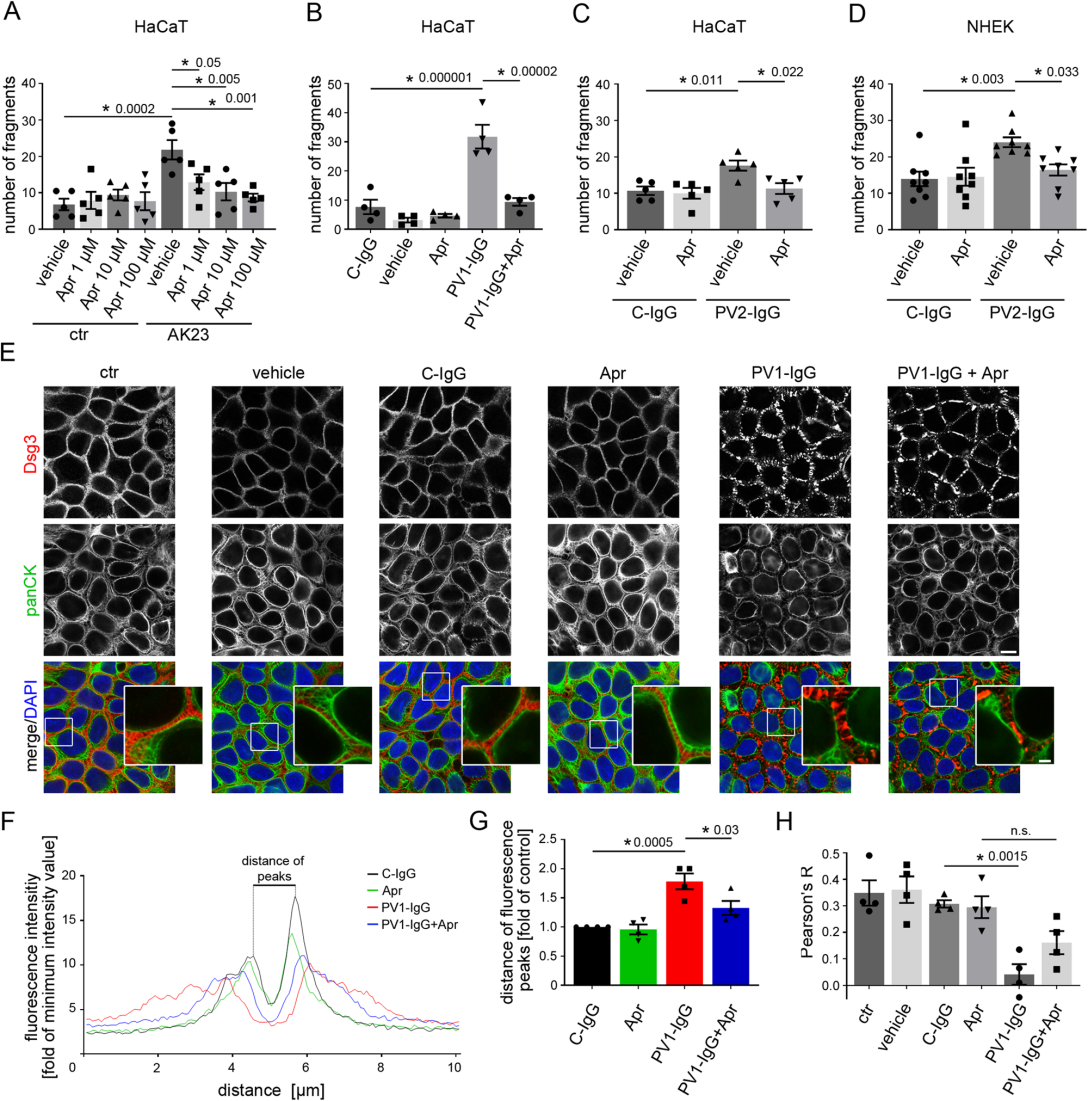
Apremilast ameliorates PV-IgG-induced loss of intercellular adhesion and keratin filament alterations. **A**–**D** Dispase-based keratinocyte dissociation assay of human keratinocytes. 1 h pre-incubation of apremilast attenuated anti-Dsg3 antibody (AK23) in a dose-dependent fashion (**A**, *n* = 5). 100 µM apremilast also ameliorated PV1-IgG- (**B**, *n* = 4) and PV2-IgG- (**C**, *n* = 5) induced loss of cell cohesion in HaCaT cells. **D** Primary keratinocytes (NHEK) show same results with PV2-IgG. (*n* = 8). **E** Immunostaining of Dsg3 and keratin filaments (panCK) in HaCaTs. Apremilast (100 µM) ameliorated PV1-induced keratin retraction but not Dsg3 depletion and fragmentation of staining. Scale bar = 10 µm. Zoom in areas marked with white rectangles. Scale bar (zoom) = 2.5 µm. Representative of *n* = 4. **F**, **G** Quantification of cytokeratin fluorescence intensity in small areas perpendicular to the respective cell border. **F** Average of keratin fluorescence intensity measured along 10 µm spanning a cell border under respective conditions. **G** Apremilast improved PV1-IgG-induced increase in distance of fluorescence peaks as a measure for retraction of the keratin cytoskeleton (*n* = 4). **H** Calculation of Pearson’s correlation coefficient. Apremilast ameliorated PV1-IgG induced loss of co-localization between keratin filaments and Dsg3 (*n* = 4). Columns indicate mean value ± SEM, *P < 0.05 (exact values are depicted in the figure). One-way ANOVA with Bonferroni correction. Source data are provided as a Source Data file.

To demonstrate the effect of apremilast on keratinocyte adhesion at concentrations used above, we performed a dose kinetic in a dispase-based keratinocyte dissociation assay. Apremilast at a dose between 1 and 100 µM significantly rescued intercellular adhesion in HaCaTs treated with AK23, a pathogenic anti-Dsg3 antibody derived from a pemphigus mouse model and PV2-IgG^49^ (Fig. 2A, S2A, B). Similarly, apremilast rescued PV1-IgG- and PV2-IgG-induced loss of intercellular adhesion in HaCaT cells and in primary human keratinocytes (NHEK) (Fig. 2B–D, S2C–E).

To investigate the effects of apremilast upon PV-IgG treatment on Dsg and keratin organization, we performed immunostaining against Dsg3 and keratin filaments in HaCaT keratinocytes (Fig. 2E). Under control conditions, Dsg3 revealed a linear staining along cell borders and a dense keratin network covered the cytoplasm. PV1-IgG-induced fragmentation of Dsg3 staining and cytokeratin staining was less intense and retracted from cell periphery in accordance to previous studies^46,47,50^. In contrast, apremilast did not rescue PV1-IgG-induced Dsg3 depletion (Fig. 2E) which was supported by Western blot analysis of HaCaT cell lysates (Figure S2F, G). However, apremilast alone induced condensation of keratin network and thickening of bundles and ameliorated PV1-IgG-induced keratin retraction (Fig. 2E–G). Quantification of cytokeratin fluorescence intensity in arrays perpendicular to the cell border revealed decreased fluorescence intensity and a significantly increased distance between fluorescence peaks in PV-IgG-treated samples which was ameliorated by apremilast (Fig. 2F, G). In addition, we calculated Pearson’s correlation coefficient to measure co-localization between keratin filaments and Dsg3. PV1-IgG diminished co-localization between these two proteins, which was improved by pretreatment with apremilast (Fig. 2E, H)^51^.

**Fig. 3:**
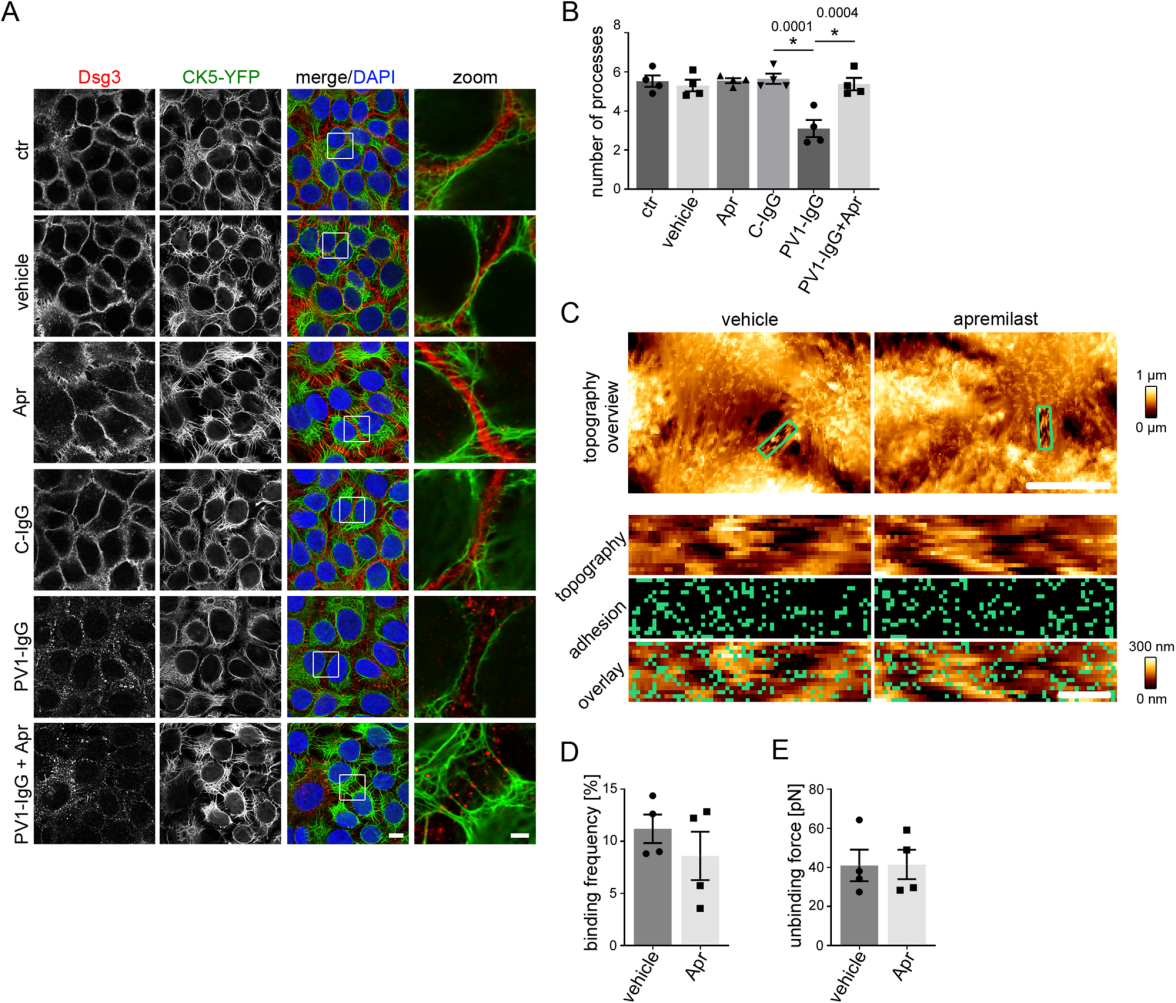
Apremilast restores PV1-IgG-induced retraction of the keratin cytoskeleton but has no effect on Dsg3. **A** Immunostaining of Dsg3 in stably expressing CK5-YFP HaCaTs. Apremilast (100 µM) prevented PV-IgG-induced retraction of the keratin cytoskeleton but not Dsg3 depletion and fragmentation of Dsg3 staining. Representative of *n* = 4. Scale bar = 10 µm. Areas for zoom in marked with white rectangles. Scale bar (zoom) = 2.5 µm. **B** Quantification of the number of keratin-bearing processes over 10 µm of the membrane. Pretreatment of apremilast restored PV1-IgG-induced decreased number of keratin-bearing cell processes (*n* = 4). **C** Topography overview images of atomic force microscopy (AFM) measurements on living HaCaTs using a Dsg3-functionalized tip revealing cell borders bridged by dense filamental structures. Scale bar = 10 µm. Small areas along the cell borders (green rectangles) were chosen for adhesion measurements. In these areas each pixel represents a force-distance-curve. In the adhesion panel each green pixel represents a Dsg3-specific binding event. Scale bar = 1 µm. **D**, **E** Quantification of AFM adhesion measurements. **D** Apremilast (100 µM) had no effect on Dsg3 binding frequency. Additionally, the unbinding force (**E**) as a measure for the single molecule binding strength was unaltered in cells treated with apremilast (8 cell borders from 4 independent experiments with 900 force-distance curves/ cell border). Bars indicate mean value ±SEM. *P < 0.05 (exact values are depicted in the figure). One-way ANOVA with Bonferroni correction (**B**), two-tailed Student’s *t* test (**D**, **E**). Source data are provided as a Source Data file.

Similar results were obtained by analyses of HaCaT cells stably expressing CK5-YFP (Fig. 3A). Intercellular contacts in HaCaTs are located in finger-like cell processes containing keratin filaments^52^. We analyzed the keratin-bearing processes over a 10 µm length of the membrane as a measure of desmosomes anchored to the keratin cytoskeleton. Apremilast completely restored the reduced number of keratin-bearing cell processes after PV1-IgG treatment (Fig. 3A, B). The specificity of staining was shown by secondary antibody control (Figure S2H).

Autoantibodies not targeting Dsg1 and Dsg3 were reported to be present in pemphigus patient^7^, from which antibodies directed against Dsc3 are pathogenic^53–55^. Thus, we checked for anti-Dsc antibodies in the PV-IgG fractions used by ELISA. Indeed, low concentration of autoantibodies against Dsc1, Dsc2 and Dsc3 were present in PV1-IgG (Table 2). To elucidate their role in loss of intercellular adhesion, we performed immunostaining for Dsc1 and Dsc3 (Fig. S3A, B). However, neither PV1-IgG nor apremilast treatment led to changes in Dsc1 and Dsc3 staining pattern indicating that apremilast does not have profound effects in these cadherins.

**Table 2:** Dsc autoantibody levels of patient-IgGs

name	Dsc1	Dsc2	Dsc3
PV1-IgG	1,7	2	1,8
PV2-IgG	1,1	1,2	1,2

Values depict OD405, fold of C-IgG and were analyzed by ELISA of purified IgG samples of pemphigus patients (IgG).

Because Dsg3 single molecule binding properties are dependent on keratins and increased cAMP levels in cardiomyocytes caused stronger adhesion of Dsg2 along cell junctions^21,56^, we performed atomic force microscopy (AFM) adhesion measurements on living HaCaT cells using Dsg3-functionalized tips to evaluate Dsg3 binding properties. Keratin filaments were readily detectable as finger-like processes along intercellular contacts in AFM topography images similar as described before (Fig. 3C, topography overview)^57^. Nevertheless, apremilast did change neither binding frequency nor binding strength referred to as the unbinding force of Dsg3 (Fig. 3C–E).

Experiments using apremilast in addition to PV-IgG were not feasible because pemphigus autoantibodies directly interfere with Dsg3 binding in living keratinocytes^52^. Thus, we repeated the experiments using PV2-IgG and performed Dsg1 adhesion measurements on murine keratinocytes which express Dsg1^56^. In accordance to previous data^48^, PV2-IgG did not lead to a direct inhibition of Dsg1 interactions and apremilast did not change Dsg1 unbinding force (Figure S3C-E). Taken together, these results indicate that apremilast strengthens cell contacts by stabilization of keratin filament anchorage rather than by affecting Dsg1 or Dsg3 binding.

### Phosphorylation of plakoglobin at S665 is induced by apremilast and required for epidermal integrity

**Fig. 4:**
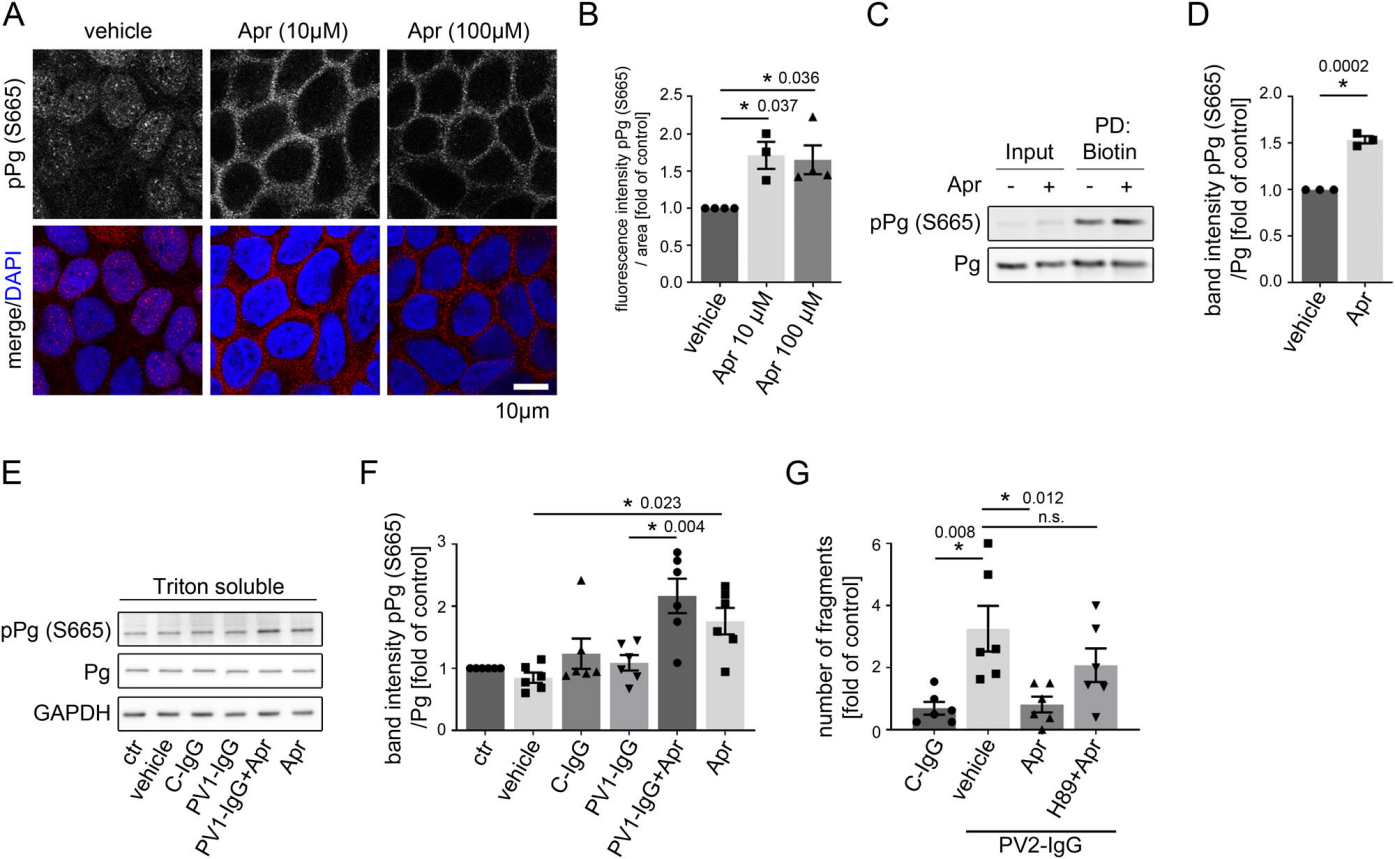
Apremilast increases phosphorylation of Pg at S665. **A** Immunostaining of phosphorylated Pg at S665 in HaCaT showed increased phosphorylation of Pg at S665 upon apremilast (10 and 100 µM) treatment. Representative of *n* = 4. Scale bar = 10 µm. **B** Quantification confirms significant stronger pPG (S665) staining along keratinocyte cell borders (*n* = 4). Bars indicate mean value ±SEM. **C** Biotinylation assay reveal higher levels of pPg (S665) at the cell membrane of HaCaT keratinocytes after apremilast (10 µM) compared to control. Representative of *n* = 3. **D** Quantification of the biotin pulldown of the biotinylation assay. Representative Western blot of HaCaT Triton soluble fraction (**E**) and quantification of band intensity of pPg (S5665) (**F)** show a significant increased phosphorylation of Pg at S665 after apremilast (100 µM) treatment. GAPDH and total Pg was used as loading control (*n* = 6). **G** Inhibition of PKA (H89) inhibited the protective effect of apremilast (10 µM) on PV2-IgG-induced loss of cell adhesion in keratinocyte dissociation assay in HaCaTs (*n* = 6). Columns indicate mean value ±SEM. *P < 0.05 (exact values are depicted in the figure). Two-tailed Student’s *t* test (**D**), One-way ANOVA with Bonferroni correction (**B**, **F**, **G**). Source data are provided as a Source Data file.

In cardiomyocytes cAMP-dependent phosphorylation of Pg at S665, a mechanism referred to as positive adhesiotropy, strengthens cell cohesion^21^. Immunofluorescence of HaCaT human keratinocytes using a phospho-specific antibody for Pg-S665^22^ showed increased phosphorylation of Pg at S665 along cell borders after treatment with 10 and 100 µM apremilast (Fig. 4A, B). This was accompanied by reduced localization of pPg (S665) in the nucleus. Similar effects were observed in intact human epidermis (Fig. S3F–H). Furthermore, higher levels of membrane-bound pPg (S665) were detected in a biotinylation assay in which membrane proteins are pulled down using membrane-impermeable biotin (Fig. 4C, D). Apremilast enhanced Pg phosphorylation at S665 significantly in HaCaT cells even when incubated together with PV1-IgG as revealed by Western blot analysis (Fig. 4E, F). Previous studies revealed that cAMP-dependent enhancement of cardiomyocyte adhesion is dependent on PKA^21^. The specific PKA inhibitor H89 diminished the protective effect of apremilast against PV2-IgG-induced loss of intercellular adhesion in dissociation assays indicating that this mechanism is also present in keratinocytes (Fig. 4G, S3I).

**Fig. 5:**
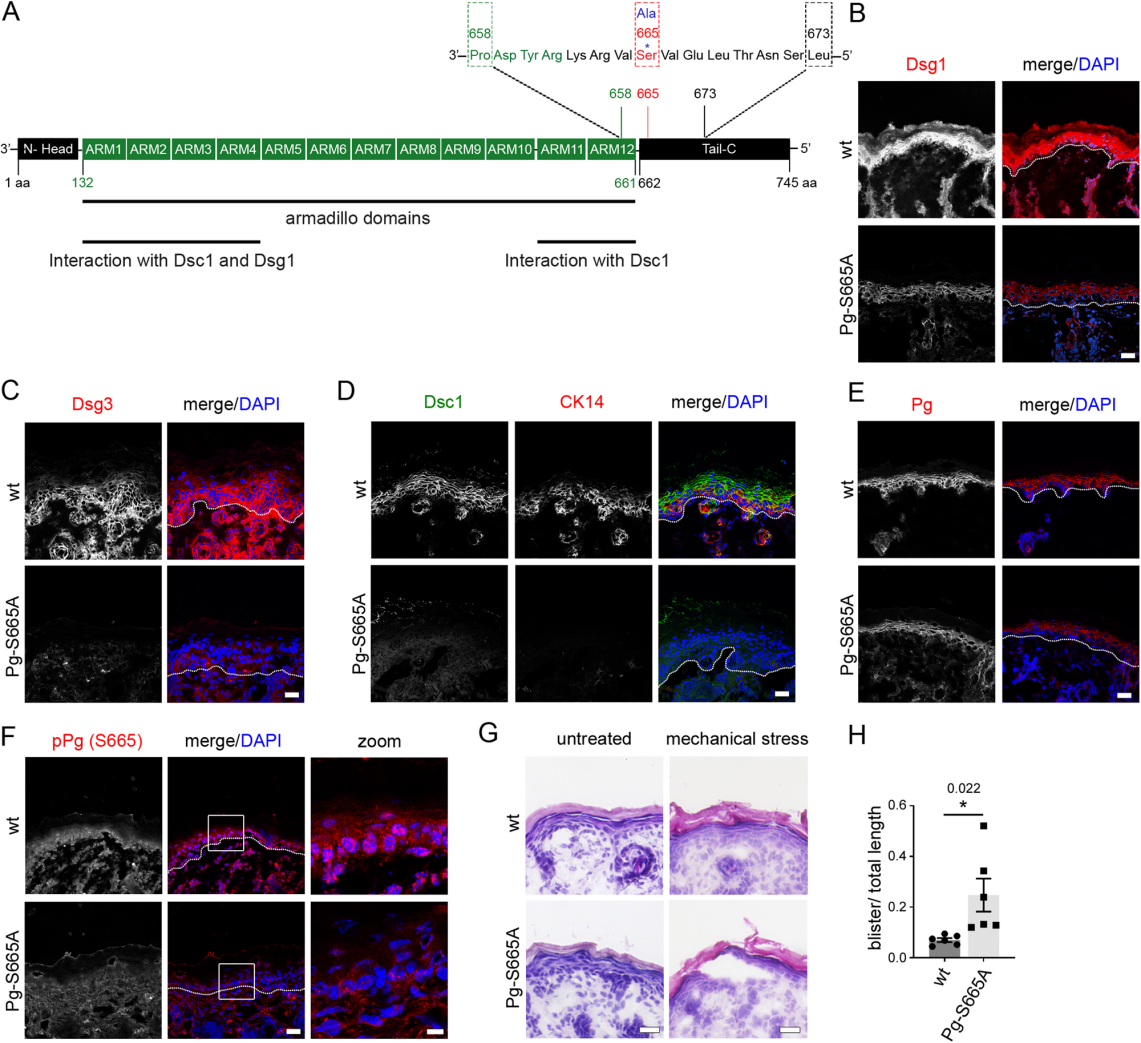
Phosphorylation of Pg at S665 is important for desmosomal adhesion in murine epidermis. **A** Introduction of S665A mutation into the plakoglobin (JUP) locus. The phospho-site of interest is located at S665 in the C-terminal tail region. **B**, **C** Immunostaining for Dsg1 (wt: *n* = 4, Pg-S665A: *n* = 7) and Dsg3 (*n* = 3) was diminished in Pg-S665A mice. **D** Dsc1 and CK14 reveal a differentiation-dependent staining pattern in wt and are almost absent in Pg-S665A mice (*n* = 5). **E** Pg is reduced along cell borders in Pg-S665A (*n* = 6) mice compared to wt (*n* = 5). **F** pPg-S665 was present in a dotted fashion along cell borders and in the nuclei in wt (*n* = 3) but not in Pg-S665A (*n* = 6) epidermis. White rectangles represent areas for zoom in. Scale bar (zoom) = 8 µm. **B**–**F** White dotted lanes represent basement membranes. Representatives of *n* ≥ 3. Scale bar = 25 µm. **G** HE staining of wt and Pg-S665A neonatal epidermis revealed no significant changes in epidermal morphology or thickness in untreated conditions. In contrast, in Pg-S665A but not in wt mice blisters occurred after exposure to mechanical stress. Representative of *n* = 6. Scale bar = 25 µm. **H** Quantification of HE after application of mechanical stress. Columns indicate mean value ±SEM. *P = 0.022. Two-tailed Student’s *t* test. Source data are provided as a Source Data file.

Next, we generated a phospho-deficient Pg-S665A *knock in* mouse model, where serine 665 was replaced by alanine (Fig. 5A; S4A–C). IF and HE stainings of the epidermis revealed normal morphology and no alterations in thickness or layer formation (Fig. 5B–G). We characterized the mouse model with regard to several desmosomal proteins to check whether their expression pattern is dependent on Pg phosphorylation at S665. Immunostaining for Dsg1, Dsc1 and Dsg3 was drastically reduced throughout all epidermal layers and appeared partly fragmented along cell borders underlining the importance of Pg and its phosphorylation at S665 for localization of desmosomal cadherins (Fig. 5B–D; Fig. S5A–C). Importantly, keratin 14 (CK14), which is predominantly present in the basal epidermal layer, was drastically rarefied (Fig. 5D; Fig. S5D), demonstrating a crucial role of Pg phosphorylation for organization of keratin filaments. Thus, we speculate that protective effects of apremilast on strengthening of the keratin cytoskeleton are dependent on phosphorylation of Pg at S665. Moreover, overall staining along cell borders of Pg was reduced indicating that the phospho-site is required for a proper turnover of Pg (Fig. 5E; Fig. S5E). As expected, pPg (S665) appeared dotted along cell borders and in the nuclei in wt epidermis, whereas it was absent in Pg-S665A epidermis (Fig. 5F).

As Dsc1 and Dsg1 were drastically altered, we wondered whether differentiation is changed in Pg-S665A-mice. However, staining for Loricrin, a cornification marker, and the tight junctions component occludin remained unaltered in the epidermis of phospho-deficient mice (Fig. S5F–I). Since E-Cadherin was also unaltered, these experiments indicate that Pg phosphorylation at S665 predominantly is required for desmosome integrity (Fig. S5J, K).

Next, we asked whether Pg phosphorylation at S665 may be required for epidermal mechanical resistance. After application of a defined shear stress Pg-S665A but not wt mice revealed microblistering of the epidermis, indicating that their overall cell adhesion is compromised (Fig. 5G, H).

### Phosphorylation of plakoglobin at S665 is required for keratin filament organization and intercellular adhesion in keratinocytes

**Fig. 6:**
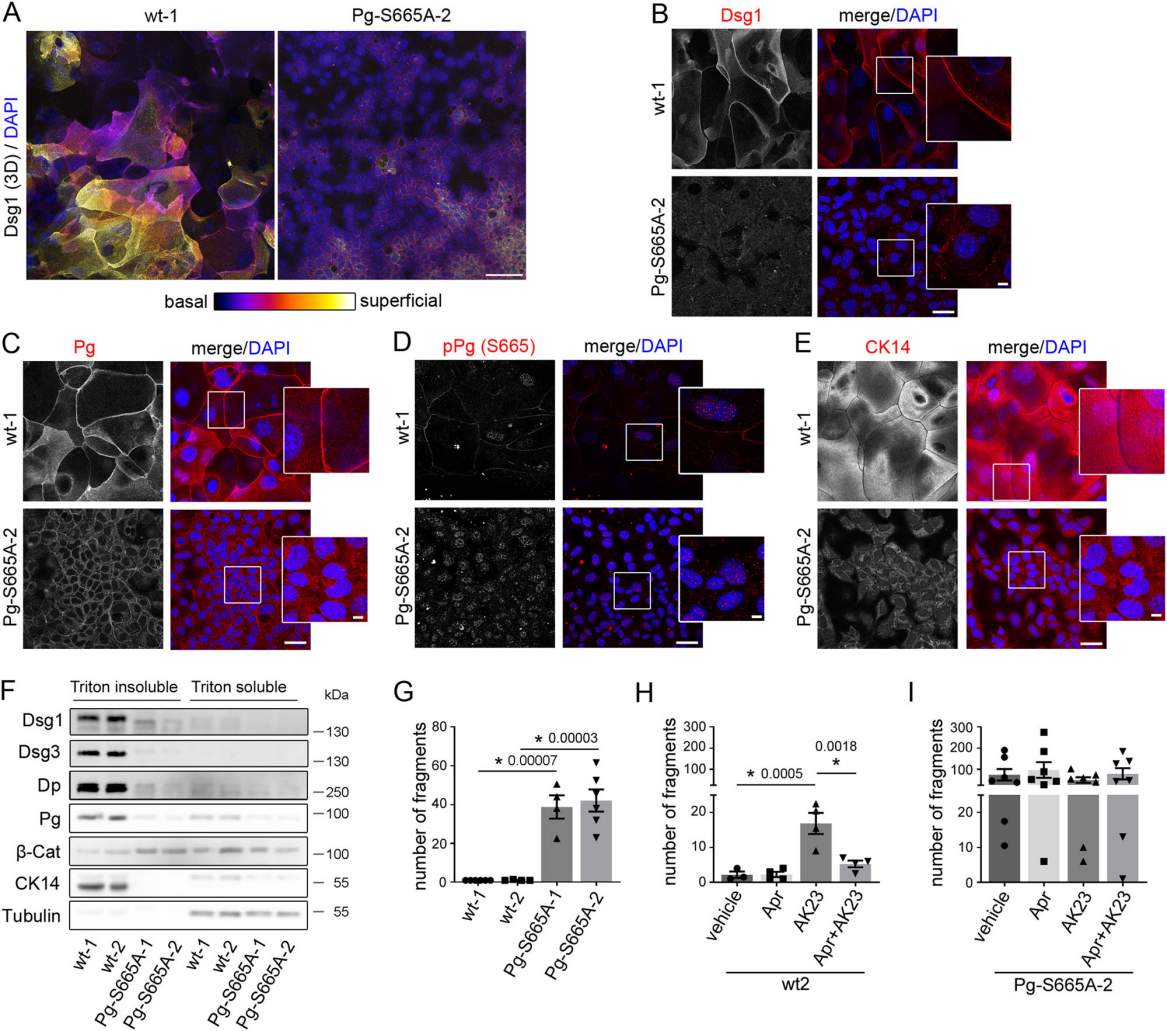
Keratinocytes phospho-deficient at Pg S665 (Pg-S665A) reveal impaired intercellular adhesion accompanied with drastic changes in desmosomal proteins and keratin filament cytoskeleton. **A** Color-encoded Z-stack of wt and Pg-S665A keratinocytes revealed multiple layers in wt and only 1-2 layers in Pg-S665A keratinocytes. Representative of *n* = 3. Scale bar = 50 µm. **A**, **B** Dsg1 was expressed primarily in the superficial epidermal layers in a linear fashion along cell borders. In contrast, Dsg1 was drastically reduced and restricted to the basal layer in Pg-S665A keratinocytes. **C** Pg was reduced and appeared less linearized in Pg-S665A keratinocytes compared to wt. **D** pPg-S665 was absent in Pg-S665A keratinocytes confirming phospho-deficiency at this site whereas it appeared dotted along cell borders and in the nuclei in wt keratinocytes. **E** The keratin cytoskeleton showed a dense network spanning the whole cell in wt keratinocytes whereas it was rarefied and drastically altered in Pg-S665A keratinocytes. **B**–**E** Scale bar = 25 µm. White rectangles depict areas for zoom in. Scale bar (zoom) = 5 µm. Representatives of *n* = 4 (**B**), *n* = 3 (**C**–**E**). **F** Triton-based separation revealed a drastic impairment of Dsg1 and Dsg3 expression in the Triton insoluble, desmosome bearing fraction in Pg-S665A keratinocytes. Similar observations were made for Dp and Pg. Importantly, keratin 14 (CK14) was almost absent in Pg-S665A keratinocytes. In contrast, β-catenin was slightly upregulated in the Triton insoluble fraction. Tubulin serves as loading control. Representative of *n* = 4. **G** Dissociation assay in wt and Pg-S665A keratinocyte cell lines showed a drastic impairment of intercellular adhesion in Pg-S665A cells (wt-1, Pg-S665A-2: *n* = 6; wt-2, Pg-S665A-1: *n* = 4). **H** Dissociation assay in wt murine keratinocytes revealed a significant fragmentation after 24 h of incubation with the pathogenic monoclonal anti-Dsg3 antibody AK23. Pre-incubation with apremilast (Apr) for 1 h ameliorated AK23-induced loss of intercellular adhesion in wt keratinocytes (*n* = 4, for vehicle: *n* = 3). In contrast, in Pg-S665A keratinocytes (**I**), which already showed drastic impairment of intercellular adhesion under resting conditions, AK23 had no additional effect on intercellular adhesion (*n* = 7). Apremilast did not restore impaired intercellular adhesion neither under basal conditions nor after AK23 incubation. Columns indicate mean value ±SEM. *P* < 0.05 (exact values are depicted in the figure). One-way ANOVA with Bonferroni correction. Source data are provided as a Source Data file.

For further investigation on the effect of Pg phosphorylation at S665 for keratinocyte adhesion we generated two murine keratinocyte cell lines from both wt (wt-1/−2) and Pg phospho-deficient mice (Pg-S665A-1/−2), respectively. In the immunostaining cells bearing the phospho-deficient mutant of Pg (Pg-S665A) were smaller in size and only barely differentiated in 2-3 cell layers as typical for wt murine keratinocytes (Fig. 6A, S6A). In accordance, differentiation-dependent expression of Dsg1 was drastically altered. Color-encoded 3D-representation of wt keratinocytes revealed that Dsg1 was mainly present in the superficial cell layers as expected from Dsg1 distribution in intact epidermis. In contrast, Dsg1 was reduced and in large parts of the cultured cells only one layer was present in Pg-S665A keratinocytes (Fig. 6A, B) indicating that Pg phosphorylation being involved in regulation of expression of differentiation-dependent desmosomal proteins in keratinocytes. Comparable to the Z-stacks above, Dsg1 and also Dsg3 staining appeared fragmented and with a reduced intensity in Pg-S665A keratinocytes (Fig. 6B, S6B–D). Further, Pg was slightly reduced along cell borders and pPg (S665) was absent along cell borders in phospho-deficient keratinocytes (Fig. 6C, D, S6E). mRNA levels of *Pg* were unaltered indicating that impaired Pg localization along cell borders is not a result of altered mRNA levels (Fig. S6F). Importantly, the keratin network which builds up a dense and delicate mesh throughout the whole cytoplasm in wt keratinocytes was substantially altered in Pg-S665A keratinocytes and appeared thinned out and irregular without a clear mesh formation (Fig. 6E, S6G). We further observed the adherens junction protein β-catenin was upregulated, while E-cadherin staining was comparable in phospho-deficient cells (Fig. S6H–K).

Similarly, Western blot analyses revealed that all desmosomal components and also CK14 were drastically reduced in Pg-S665A cell lines in both the triton -soluble and -insoluble fraction, whereas β-catenin was up-regulated in the triton insoluble, desmosome containing fraction (Fig. 6F, S7A–G).

Next, we investigated the effect of Pg-S665A on intercellular adhesion. Pg-S665A keratinocytes revealed a significantly and drastically impaired intercellular adhesion in dissociation assays (Fig. 6G, S7H). We asked whether a pathogenic pemphigus autoantibody such as AK23 would have additional effects on cell adhesion in Pg-S665A keratinocytes and if apremilast would rescue cell adhesion. We applied AK23 and apremilast for 24 h in wt and Pg-S665A keratinocytes and performed dissociation assays. In wt cells, AK23 led to fragmentation and thus loss of intercellular adhesion which was significantly reduced by apremilast (Fig. 6H, S7I). However, both AK23 and apremilast did not affect intercellular adhesion in Pg-S665A keratinocytes (Fig. 6I, S7J).

**Fig. 7:**
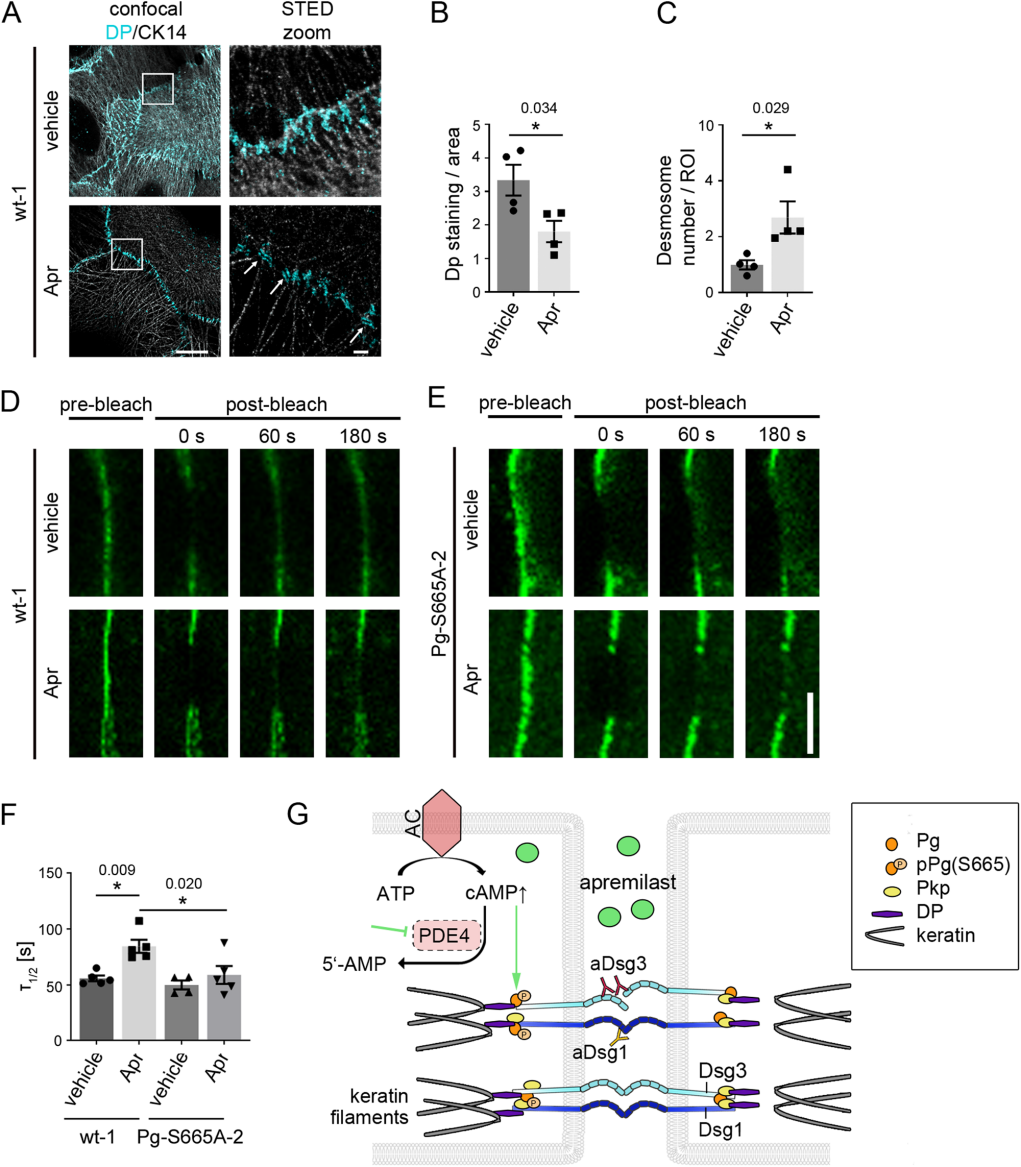
Apremilast regulates desmoplakin recruitment and Dsg3 membrane availability. **A** Representative immunostaining of wt murine keratinocytes incubated with apremilast for 2 h and stained for desmoplakin (Dp) and cytokeratin 14 (CK14). Under control conditions, Dp was present at linear streaks along the cytokeratin filament at cellular contact points. In contrast, after apremilast (10 µM) treatment, Dp was present in desmosomes (white arrows). Scale bar = 10 µm. Zoom in areas marked with white rectangles. Scale bar (zoom) = 1 µm. Representative of *n* = 4. **B**, **C** Quantification of Dp staining. Dp-covered area is significantly reduced after apremilast treatment compared to control cells. In contrast, number of desmosomes/ROI was elevated. **D**, **E** FRAP on murine keratinocytes transfected with Dsg3-GFP. **D**, **F** In wt murine keratinocytes, apremilast (10 µM) lead to enhanced recovery halftime (τ1/2). Scale bar = 5 µm. **E**, **F** In contrast, no changes in τ were present in Pg-S665A keratinocytes (*n* = 5, for Pg-S665A vehicle: *n* = 4). **G** Schematic of mechanisms of protective cAMP signaling in pemphigus. Apremilast prevents PV-IgG-induced loss of intercellular adhesion. Autoantibodies directed against desmogleins induce uncoupling of keratin filaments from the desmosomal plaque. Increase of intercellular cAMP by the phosphodiesterase 4- (PDE4) inhibitor apremilast ameliorates this effect by PKA-dependent phosphorylation of Pg at S665. Columns indicate mean values ±SEM. *P < 0.05 (exact values are depicted in the figure). Two-tailed Student’s *t* test (**B**, **C**), one-way ANOVA with Bonferroni correction **(F)**. Source data are provided as a Source Data file.

Taken together, we observed that Pg phosphorylation at S665 is crucial for proper adhesive function of desmosomes and organization of the keratin cytoskeleton of keratinocytes. Therefore, we further investigated how cAMP regulates desmosome turn-over. Ca^2+^ switch assays to synchronize desmosome assembly as performed previously^15^ did not indicate differences in overall desmosome assembly in the time courses of 8 h and 24 h in presence or absence of apremilast, respectively (Figure S8A, B). We further concentrated on later steps of desmosome assembly and performed STED microscopy in murine wt keratinocytes cells after co-labelling of CK14 and Dp. In controls, Dp stretched out along the terminal ends of keratin filaments adjacent to the cell membrane (Fig. 7A). In contrast, after incubation with apremilast Dp localization was more confined to proper desmosomal plaques (indicated by arrows). In line with this, Dp staining area was reduced after apremilast treatment, whereas the number of desmosomes along cell borders increased significantly (Fig. 7B, C). Comparable experiments were not feasible in Pg-S665A keratinocytes due to their severely impaired localization of desmosomal components at cell borders as described above. These data indicate that apremilast modulated Dp recruitment to nascent desmosomal contacts. Finally, to test whether this would affect desmoglein turnover at the membrane and to address the role of Pg phosphorylation in this process, FRAP was performed after transfection of cell lines with Dsg3-GFP (Fig. 7D). Halftime of fluorescence recovery (τ_1/2_) was reduced after apremilast incubation in wt but not in Pg-S665A keratinocytes whereas overall Dsg3 mobility was comparable under all conditions (Fig. 7D-F, S8C). These data are in line with reduced Dsg3 membrane availability in bleached areas in consequence to enhanced Pg-mediated cytoskeletal anchorage of Dsg3.

## Discussion

Pemphigus therapies rely on therapeutic strategies to suppress the immune system and are associated with severe side-effects. In contrast, no medication is available that directly targets adhesion of human keratinocytes. Therefore, we characterized whether apremilast-induced protective cAMP signaling may be effective to prevent acantholysis in PV and investigated the underlying mechanisms. We found that apremilast prevented PV-IgG-induced epidermal blistering in human epidermis ex vivo and in a murine pemphigus mouse model in vivo. In human epidermis, PV-IgG-induced loss of keratin filament anchorage was rescued by apremilast. These results were supported in vitro where apremilast abrogated PV-IgG-mediated loss of keratinocyte adhesion and blocked keratin filament reorganization, which is a morphological hallmark in pemphigus present in both patients’ skin lesions and human skin models^45,58^. We established a Pg-S665-phospho-deficient mouse model and found that phosphorylation of Pg at S665 is crucial for maintenance of epidermal integrity and keratinocyte adhesion.

### Apremilast enhances cAMP signaling in keratinocytes and stabilizes keratinocyte adhesion in pemphigus

cAMP increase regulates cadherin-mediated adhesion in various tissues. For instance, for VE-cadherin, cAMP increase was reported both to stabilize VE-cadherin at the cell membrane and to contribute to proper barrier function of the endothelium^59^. Further, cAMP increase in cardiomyocytes led to a phenomenon referred to as positive adhesiotropy and had a direct effect on adhesive strength of cardiac desmosomes. Adrenergic signaling induced redistribution of Dsg2 towards cell borders of cardiomyocytes and strengthened intercalated discs on the ultrastructural level^21,22^.

Here, we show that apremilast via protective cAMP signaling in keratinocytes is a promising approach to target loss of desmosomal adhesion in pemphigus patients. Apremilast prevented blister formation in a human ex vivo model as well as in a murine passive transfer PV model in vivo. In line with this, we reported previously that cAMP increase induced by the combination of the adenylate cyclase (AC) activator forskolin with the phosphodiesterase inhibitor rolipram (F/R) similar to the β-sympathomimetic isoprenaline abolished loss of desmosomal adhesion caused by pemphigus autoantibodies in vitro and in a passive immune transfer mouse model in vivo^20^.

Several signaling mechanisms are critically involved in pemphigus pathogenesis, including p38MAPK, Erk, Src, PKC and Ca^2+^ ^8–10,12^. These signaling pathways are dysregulated upon autoantibody binding and drive loss of intercellular adhesion. Accordingly, modulation of such signaling is protective in different models of pemphigus. However, a therapeutic strategy can rarely be derived from these investigations as mediators targeting the respective signaling pathways systemically in patients most likely would cause severe side effects. Nevertheless, a recent study identified novel treatment targets including MEK1, TrkA, PI3Kα, and VEGFR2 in pemphigus using an unbiased library approach, revealing the importance of therapies in pemphigus directly targeting keratinocytes^17^. Therefore, since apremilast is clinically approved to be effective and safe in psoriasis patients^33^, we further characterized the mechanisms underlying the protective effects of apremilast on keratinocyte adhesion.

Strikingly, apremilast abolished keratin filament reorganization, which is an ultrastructural hallmark in both pemphigus patients’ skin lesions and human skin models^45,58^ (summary Fig. 7G). In our experiments, apremilast abrogated retraction of endogenous keratin filaments in cultured cells and intact epidermis ex vivo similar to filaments in cells overexpressing CK5-YFP. These data support previous findings that protective effects in pemphigus models were paralleled frequently by preventing keratin retraction^46,60^. Moreover, keratins critically contribute to the proper adhesive function of desmosomal cadherins and thus to the integrity of desmosomes^48,56,61^.

In contrast to F/R and isoprenaline^20^, treatment with apremilast did not affect Dsg depletion both in vitro and in human skin ex vivo. This discrepancy may be explained by distinct amounts of cAMP in keratinocytes treated with either apremilast or F/R but may also be due to different downstream targets or compartmentalization of cAMP^62^. In line with this observation, keratin retraction and Dsg3 depletion independently contribute to loss of intercellular adhesion in pemphigus^47^. We observed that halftime of recovery of Dsg3 fluorescence in FRAP experiments was increased after apremilast treatment which may be interpreted as stabilization of Dsg3-containing desmosomal precursors in the cell membrane. In addition, apremilast enhanced incorporation of Dp into forming desmosomal plaques, a process critical for desmosome assembly^63,64^. Taken together, cAMP appears to drive keratin filament anchorage of desmosomes via recruitment of Dp, which also may explain strengthening of keratin filament insertion as visible by TEM.

### Plakoglobin S665 phosphorylation is critical for keratinocyte adhesion and epidermal integrity

In cardiomyocytes, positive adhesiotropy was dependent on the desmosomal plaque protein Pg and its phosphorylation at S665 via protein kinase A (PKA). In accordance, overexpression of a phospho-deficient Pg-mutant (Pg-S665A) abolished positive effects of cAMP increase on cardiomyocyte adhesion^21^. Similarly, in keratinocytes PKA contributes to protective cAMP signaling in pemphigus and H89 abolishes the protective effects of apremilast^20^. Because we observed that Pg is also phosphorylated at S665 in keratinocytes when cAMP levels were increased by incubation with apremilast both in vitro and ex vivo, we further investigated the role of Pg S665-phosphorylation for keratinocyte adhesion. To this end, we established a previously unreported Pg-S665A-phospho-deficient *knock-in* mouse model and derived murine keratinocytes from these mice.

Although mice bearing the Pg-S665A mutation were viable and showed no histologic epidermal alterations, epidermis was fragile and the desmosomal composition of keratinocytes was critically altered. The pemphigus antigens Dsg1 and Dsg3 were downregulated and the keratin cytoskeleton was severely compromised. This was recapitulated in cultured keratinocyte cell lines in which phospho-deficiency of Pg at S665 drastically impaired intercellular adhesion.

### Apremilast as a new treatment paradigm to stabilize keratinocyte adhesion in skin diseases

Apremilast is a well-established therapeutic approach in psoriasis^32–34^. However, the mechanisms underlying the clinical effects of apremilast were up to now attributed to modulation of the immune system^32^. For pemphigus, a recent case report in which a chronic, non-responsive PV patient was successfully treated with apremilast underlines the clinical importance of apremilast in treatment of skin diseases^36^. Here, we report that PDE4-inhibition by apremilast not only affects the immune system, but also leads to a protective cAMP increase and strengthens intercellular adhesion in keratinocytes. Importantly, apremilast at a concentration of 1 µM abolished blister formation in vivo in a neonatal pemphigus mouse model. This is important as a well-established drug administration of 2×30 mg p.o./ day leads to comparable plasma concentrations without severe side effects (https://www.ema.europa.eu/en/documents/product-information/otezla-epar-product-information_de.pdf).

Interestingly, it was reported recently that cAMP levels are increased and AC9 was upregulated in psoriatic skin models^65^ and that β2-adrenergic receptors are downregulated in psoriatic skin lesions^66^. Therefore, it was postulated that cAMP may contribute to psoriatic hyperproliferation. However, considering our observation that increased cAMP may serve as a rescue pathway in pemphigus, the findings may also be interpreted in the way that positive adhesiotropy induced by adrenergic signaling is a general rescue pathway in inflammatory skin disorders. This hypothesis is further strengthened by the fact that apremilast is an effective and safe therapeutic mediator in psoriasis^67,68^ which may also have direct effects on keratinocytes. Various case reports showed a simultaneous occurrence of PV and psoriasis^69,70^ and an elevated risk for pemphigus was suggested for patients suffering from psoriasis^71^, suggesting a similar predisposition for both diseases by dysregulated cAMP signaling in keratinocytes. Furthermore, apremilast was beneficial in a clinical study on palmoplantar keratoderma^72^, which at least in part may also be caused by effects of apremilast on keratin filament organization in keratinocytes.

Taken together, our data provide a novel therapeutic approach in pemphigus to enhance protective cAMP signaling in keratinocytes through apremilast. In addition, we demonstrate an to our knowledge unreported mechanism involved in desmosome regulation via cAMP-induced Pg phosphorylation at S665, which strengthens Dp recruitment and intermediate filament anchorage of desmosomes (Fig. 7G). Therefore, we propose a new paradigm according to which apremilast may be effective to treat skin diseases, including psoriasis and pemphigus not only by modulation of the immune system but rather also via direct effects on keratinocytes.

## Methods

This studies comprise animal experiments and experiments with normal human keratinocytes and cadaver skin. All studies comply with all relevant ethical regulations. Animal maintenance and breeding are covered by an animal breeding proposal (ethical board of Regierung von Oberbayern, ROB 55.2-2532.Vet_02-19-172). Animal experiments were approved by an ethical board of Regierung von Oberbayern (Vet 02_21_205). Isolation of normal human keratinocytes and respective experiments were approved by medical ethical committee of the Eberhard Karls University Tübingen (ethical approval: EK318-21). Experiments with cadaver skin were performed in context of the body donor program of the anatomy department of LMU Munich. Body donors gave their written and informed consent for the use of skin samples for research purposes. The local ethical committee of LMU Munich gave their confirmation that no further approval for the experimental protocol is required.

For extended information please refer to Suppl. Material.

### Generation of *Knock-in* mouse model for Jup S665A mutation

To introduce the S665A mutation into the plakoglobin (JUP) locus, a targeting vector (I143.4 TV, Figure S4A) containing two homology regions of 6,6 kb (long homology arm, LA) and 2,86 kb (short homology arm, SA) framing the neomycin resistance cassette was generated.

Both, LA and SA, were a product of a PCR where C57Bl/6 N BAC DNA was used as a template. Thus, a 2,86 kb NotI - EcoRV fragment spanning exon 9 (E9) and an adjacent 6,6 kb SbfI - AscI fragment containing the genomic region of JUP from exon 10 (E10) to exon 14 (E14) were amplified. Both homologous arms were subcloned on either side of a neomycin (Neo) resistance cassette, introduced previously into pBS-KS vector bone. The Neo cassette was composed of two FRT sites integrated on both ends of the coding sequence for Neo resistant gene expressed under the control of Simian Virus 40 (SV40) promoter. The Neo selection marker was inserted in a less conserved region within the intron 9, in an area about 130 nucleotides upstream of E10. The S665A mutation was introduced within the LA, about 500 bps downstream of the insertion site of the neomycin cassette. More specifically, the mutation is located in exon E11 and it is caused by a transition of the first T to G in the TCT codon, encoding for serine (Ser) in CDS position 1993. This resulted in generation of GCT nucleotide triplet which codes for alanine (Ala) (Figure S4A). To incorporate the mutation, an overlay-extension PCR was performed. Hence, two PCRs were run in parallel using different primer pairs (pair 1: I143.4/I143.6 and pair 2: I143.5/I143.7; Table S1). The resulting PCR products served as a template for a third PCR with the primers set I143.4/I143.5 (Table S1 and Figure S4B). A PCR fragment, spanning E10 and mutation-carrying E11 was amplified and subsequently cloned into the LA, within the I143.4 TV targeting vector, by using SbfI and BstEII restriction sites. As a final step, to verify the integrity of the vector bone and the newly inserted regions, in addition to the restriction analysis, a complete sequencing of the key regions within the targeting vector was done.

#### Detection of homologous recombination in embryonic stem cells

C57BL/6N-derived embryonic stem (ES) cells were transfected with I143.4 TV targeting vector. This generated heterozygous cells containing both a targeted allele carrying the plakoglobin/JUP mutation, and a wild type (WT) allele (Fig. S4B). Prior to the electroporation of ES cells, the targeting vector was linearized with the NotI restriction enzyme. For selection and maintenance of the ES cells transfected with the vector carrying a Neo resistance gene, G418 was added at a final concentration of 0,2 mg/ml. Following 8 days of selection, a total of 192 clones were picked and tested to verify a successful homologous recombination. For this purpose, a screening PCR was established, where a newly constructed primer pair (I143.9/518LRPCR2) was designed to align only to the targeted allele, but not to the WT allele (Table S1 and Fig. S4B). Additionally, the primer set was selected so that it gives an amplicon only when a recombination event occurs in the targeted locus, but not in any other random locus. Therefore, the 518LRPCR2 primer specifically binds to the vector and not to the genome; within a region of the neomycin cassette. In contrast, the I143.9 primer aligns to the genome only, more precisely, to a sequence that is not part of the targeting vector. Thus, if a signal was detected, an association between the vector and the Jup locus could be validated.

To test the specificity of the primers, a control vector (I143.2 CV) was generated, which was later used as an internal positive control for the screening PCR. The vector contains an elongated SA homology region, which holds 50 bp of extra genomic sequence that is not present in the I143.4 TV targeting vector and is used as a template to which a I143.9 primer is selectively aligning. In addition to the screening PCR analysis, the homologous recombination in the ES was confirmed by sequencing and Southern blot analysis.

#### Southern blot analysis

The validation of positive clones carrying correct homologous recombination with the Jup locus was achieved by Southern blot analysis.

DNA from individual clones was digested with the restriction enzyme NsiI, the location of the sites are shown in Fig. S4B, confirming that, compared to the WT, the targeted allele (TG) has additional NsiI restriction sites, specifically localized within the neomycin cassette. NsiI-digested genomic DNA was then analyzed using [α*-*32P] dCTP DNA 3’ probe that corresponds to the genomic sequence downstream of the incorporated mutation (Fig. S4B). The 3’ external probe (516 bp in length) was generated by PCR using the primer combination I143.21/I143.22 (Table S1) and genomic DNA as a template. The probe was located approximately 3,4 kb downstream of E14 and about 880 bp downstream from the end of LA homologous arm. After gel extraction and purification, the probe was used to identify the complementary DNA fragment of interest. Due to the size of the Neo cassette, the TG allele is larger than the WT allele, therefore, the size of the restriction fragment detected by the probes was 9,9 kb. In contrast, presence of a signal corresponding to 8.6 kb, confirmed the existence of a WT allele.

#### Detection of S665A mutation in the ES cell genome

To test for the presence of the mutation in ES cells, a region surrounding the mutation was amplified using a forward primer (I143.4), specific for region in intron 9, and a reverse primer (I143.5), binding to intron 11 (Table S1 and Fig. S4B). Designed primers yielded a PCR amplification product with a size of 1094 bp in both, TG- and the WT- allele. The 1094-bp sized fragment was purified from a gel and subsequently sequenced with either of the primers used for its amplification.

#### Blastocyst injection and breeding

Selected ES cell clones were injected into blastocysts from grey C57BL/6 N mice. The surviving blastocysts were then transferred into the uterus of pseudo-pregnant animals (3 CD-1 foster mice). This resulted in the birth of chimeric heterozygous transgenic mice, which were then backcrossed with germ-line Flp expressing mice (“Flp deletors”). Upon this breeding scheme, the FRT-flanked Neo was excised by Flp-FRT recombination, initiated by the Flp recombinase delivered from the “Flp deletors”. This led to the establishment of black offspring carrying the targeting construct with a deleted Neo cassette.

#### Genotyping of constitutive JUP Ser665Ala KI mutant

For genotyping of pups from the F1 and F2 generation, genomic DNA extracted from tail or ear tissue was use as a template for a series of different PCRs.

By using the I143.27/I143.28 primer combination (Table S1 and Fig. S4C), the samples were screened for the Flp-mediated deletion of the Neo cassette, which is shown by the presence of the remaining FRT site. Positive animals are germline transmitters of the targeted Jup allele after deletion of the Neo cassette. The predicted amplicon for WT allele is 568 bp in size, while for the targeted deleted Neo (TG del-neo), a 686 bp fragment is expected.

To identify offspring that still contained the Neo cassette, the primer combination Neo.MP1/Neo.MP5/Neo.MP6 (Table S1 and Fig. S4C) was used for screening. In contrast to Neo.MP1, which binds to a sequence from the Neo cassette and to a region within chromosome 3, Neo.MP5 and Neo.MP6 are unique and align to either of the above mentioned regions. Therefore, whereas both primers of the set Neo.MP1/Neo.MP6 bind to regions within the Neo cassette (expected product of 512 bp), the primer combination Neo.MP1/ Neo.MP5 align to regions within the chromosome 3 (PCR product with expected size of 380 bp, used as an internal control). Additionally, the mice were screened for the presence of the Flp recombinase using the primer combination SD24/SD25, which results in a 568 bp-sized PCR fragment. Finally, to test for the presence of the S665A mutation in Jup Ex 11, amplification of a region (875 bp in size) spanning the mutation was screened by using I143.25/I143.26 primer set (Table S1 and Fig. S4C).

For characterization of the mouse model, neonatal animals were sacrificed and skin was taken for immunofluorescence staining and Western blot analysis. Mechanical stability was determined by application of a defined mechanical stress by pinching the mice skin 3 times (Nikolsky phenomenon).

### Mice maintenance

Mice were maintained in breeding facility (Max von Pettenkofer Institut, LMU Munich) under approved animal breeding proposal of an ethical board of the Regierung von Oberbayern (ROB 55.2-2532.Vet_02-19-172). As cages the IVC System „Seal-Safe“ (Tecniplast, Hohenpeißenberg, Germany) were used and mice were maintained at 22 ± 1,5 °C and a humidity 50 ± 5% with a 12 h/ 12 h dark/light cycle.

### Neonatal pemphigus mouse model

For the pemphigus mouse model neonatal BALB/cAnNCrl (2d old) were used. Animal experiments and protocols were approved by an ethical board of Regierung von Oberbayern (Vet 02_21_205). Neonatal mice were separated from mother and maintained at 37 °C. Mice were fed every 2 h and were monitored using a score sheet confirmed by an ethical board of the Regierung von Oberbayern (Vet 02_21_205). Mice were injected subepidermal in the backskin with 50 μl of either control-IgG (derived from healthy volunteer) or PV1-IgG^13,20^. Mice were preinjected with 50 μl vehicle or apremilast (1 µM) 2 h before receiving the IgG fractions. After 10 h mice were sacrificed by decapitation and a defined shear stress was applied by pinching (similar to Nikolsky sign) and skin samples were collected. Every 400 μm 1 section was checked for the presence of intraepidermal blisters after staining with 1% toluidine blue solution. Representative cryosections were subjected to HE staining.

### Cell culture and test reagents

Experiments were performed in HaCaT immortalized human keratinocytes^73^, murine keratinocytes or normal human keratinocytes (NHEK). NHEK were generated at Universitäts-Hautklinik Tübingen with written and informed consent from the donors^74^. The procedure was approved by the medical ethical committee of the Eberhard Karls University Tübingen (ethical approval: EK318-21).

For some experiments, HaCaT cells stably transfected with keratin5-YFP (kind gift of Reinhard Windoffer and Nicole Schwarz, Institute of Molecular and Cellular Anatomy, RWTH Aachen University) were used. HaCaTs were grown in Dulbecco’s Modified Eagle Medium (Thermo Fisher Scientific, Waltham, Massachusetts) supplemented with 10% FCS (Biochrom, Berlin, Germany), 50 U/ml penicillin and 50 g/ml streptomycin (both AppliChem, Darmstadt, Germany) and NHEKs were cultured in epidermal keratinocyte medium (CnT 0.7, Cellntec, Bern, Switzerland). NHEKs were grown in low calcium (Ca^2+^) (0.06 mM) until confluency and switched to 1.8 mM Ca^2+^ 24 h prior experiments. Experiments were performed between passage 3 and 6. All human keratinocytes were cultured at 37 °C in a humidified atmosphere of 5% CO_2_.

Murine keratinocytes from JUP S665A mice strain were generated using a well-established protocol^61,75^. In short, epidermis of neonatal mice were removed using Dispase II (Sigma-Aldrich, Munich, Germany) and epidermal cells were isolated by treatment with Accutase (Sigma-Aldrich) for 1 h. Cells were seeded in complete FAD media (0.05 mM CaCl_2_, PAN Biotech, Aidenbach, Germany) on flasks coated with collagen I (rat tail; BD Bioscience, New Jersey, US). Keratinocytes immortalize spontaneously after 10-15 passages. Subsequently cells were grown under previous mentioned conditions and switched to high Ca^2+^ (1.2 mM) 48 h before experiments were started^75^. For assembly experiments Ca^2+^ (1.2 mM) was added at a given time before experimental procedure together with apremilast (8 h, 24 h). Cells were maintained in a humified atmosphere with 5% CO_2_ at 35 °C.

### Purification of patients IgG fractions and AFM proteins

Sera samples of healthy volunteers and PV patients were used with informed and written consent under approval of the local ethic committee of University of Lübeck (number: AZ12-178). The phenotype of the disease was clinically and histopathologically characterized and the antibody profile was measured using Dsg1 and Dsg3-ELISA (Euroimmun, Lübeck, Germany). Presence of anti- anti-Dsc1, anti-Dsc2, anti-Dsc3 antibodies were checked by ELISA (Marburg, Germany). All titers are given in Tables 1 and 2.

IgG-fractions were purified using protein A affinity chromatography^13,76^. Serum was incubated with protein A agarose (Merck, Darmstadt, Germany) in purification columns for 2 h, washed with PBS, eluted with 20 mmol/l sodium citrate solution, neutralized in 2 mol/l sodium carbonate solution and concentrated in a filter unit (Amicon® Ultra-4, Merck, Darmstadt, Germany) at 19000 g for 20 min. Afterwards the antibodies were buffered in PBS and stored at −20 °C.

AK23, a pathogenic monoclonal anti-Dsg-3-antibody produced in a mouse model, was bought from Medical & Biological Laboratories Co., Ltd. (Nagoya, Japan) and used at a concentration of 75 µg/ml.

Dsg3-Fc and Dsg1-Fc construct, comprising the full extracellular domain of Dsg3 or Dsg1, respectively, were expressed in Chinese hamster ovary cells. After reaching confluency, supernatants were collected and recombinant proteins were isolated by protein A agarose affinity chromatography (Life Technologies/ThermoFisher scientific, Waltham, USA; details please refer to purification of Antibodies). Coomassie staining and Western blot analysis were performed to test purity.

### Measurement of cAMP levels

The cAMP enzyme-linked immunosorbent assay (EIA, CA-200, Sigma-Aldrich, St. Louis, MO, USA) was used to evaluate the levels of intracellular cAMP levels. Cells were incubated, washed and lysed by 0.1 M HCl. Afterwards, the EIA was performed according to the manufacturer’s manual. The intensity of color change was measured by a spectrophotometer (Tecan Plate Reader with Magellan software version V7.2, Männedorf, Switzerland).

### Keratinocyte dissociation assay

For keratinocytes dissociation assay confluent keratinocyte monolayers were removed from well bottom after respective incubation using 200 µl Dispase II (Sigma Aldrich, St. Louis, Missouri). In murine keratinocytes Dispase II solution was supplemented with 1% of collagenase I (Thermo Fisher Scientific, Massachusetts, US). Subsequently, monolayers were subjected to a defined mechanical stress. Resulting fragments are an inverse measure for intercellular adhesion and were counted either manually or using ImageJ (NIH, Bethesda, Maryland).

### Ex-vivo pemphigus skin model

Human ex vivo pemphigus model was performed with cadavers of the human body donor program without history of skin diseases from the Institute of Anatomy, Ludwig-Maximilians-University (LMU) Munich, Germany^45^. Experiments and protocols are covered by the body donation program of the Institute of Anatomy, LMU Munich, Germany. Written informed consent was obtained from body donors for the use of research samples. Local ethical commission of LMU confirmed that no further ethical approval is required for experimental protocol. Age and sex of body donors are listed in table S2. Skin samples were taken from the area covering the deltoid muscle from body donors who had died no longer than 24 h before and thus material cannot be made accessible to other researchers. A 30.5 G needle (B.Braun, Melsungen, Germany) was used to inject either 50 µl Apremilast in DMSO or DMSO and after 1 h of pre-incubation another 50 µl of either PV- or C-IgG. Samples were incubated in Dulbecco’s modified Eagle’s medium (DMEM) in a humidified atmosphere of 95% air and 5% CO_2_ at 37 °C for 24 h. After incubation a defined shear stress was applied using a rubber. Subsequently, specimens were either embedded in Tissue-tec (Leica, Wetzlar, Germany) for cryosectioning or in glutaraldehyde for electron microscopy analysis. All data shown in Fig. 1 represent skin from the area the shear stress was applied.

### Electron microscopy

The treated skin pieces, 24 h after incubation, were dissected into small pieces (approximately 2 mm) and fixed by immersion in 2.5% glutaraldehyde in PBS for 1 h at room temperature. The samples were either transferred to PBS for storage at 40 °C or subsequently processed as described in Egu et al., 2017^77^. To do so, specimen were taken through three washes of PBS buffer and post-fixed in 2% osmium tetroxide for 3 h. This was followed by successive washings in ascending ethanol series and finally cleared in propylene oxide. The samples were finally embedded in EPON 812 resin (SERVA Electrophoresis GmBH, Heidelberg, Germany), and cured as per the company’s recommendation. The resulting blocks were sliced at 60 nm thickness using ultramicrotome (Reichert-Jung Ultracut E, Optische Werke AG, Vienna, Austria) with a diamond knife (DiATOME Electron Microscopy Sciences, Hatfield, PA). Silver shining sections were harvested using 150 mesh copper/rhodium grids (Plano GmbH, Wetzlar, Germany). Finally, sections were contrasted using uranyl acetate and lead citrate. Images were captured at 4000x and 12,500x magnification with a Libra 120 transmission electron microscopy (Carl Zeiss NTS GmbH, Germany) equipped with a SSCCD camera system (TRS, Olympus, Tokyo, Japan).

#### Ultrastructural quantification

Electromicrographs were taken along the cleavage plane of pemphigus vulgaris in the deep layer of the epidermis. Accordingly, desmosomes between adjacent basal keratinocytes as well as those between basal and suprabasal keratinocytes were considered in the evaluation. Images were captured at 4000x magnification, and for each set of experiment 20 −30 images and ~100 desmosomes were evaluated per condition using ImageJ software (Wayne Rasband; https://imagej.nih.gov/ij/). Measurement of desmosome size and number as well as quantification of split desmosomes and retracted keratin filaments in the vicinity of blister formation was performed as previously described^78^.

### Cell lysis and Western blotting

For protein analysis cells were washed and lysed in 60 µl SDS-lysis-buffer (25 mM HEPES, 2 mMol EDTA, 25 mM NaF and 1 % sodiumdodecylsulfate, pH 7.6, protease inhibitors). For some experiments cells were incubated with extraction buffer (0.5% Triton-X-100, 50 mM MES, 25 mM EGTA, 5 mM MgCl_2_, pH = 6.8, protease inhibitors Aprotenin, Pepstatin, Leupeptin, and phenylmethylsulfonylfluoride) for 20 minutes on ice, scraped and afterwards centrifugated at 19.000 g to split triton-soluble non-cytoskeletal bound from the cytoskeletal bound non-soluble proteins. Supernatants containing the Triton-soluble fraction were collected and pellet was resuspended in SDS-lysis-buffer.

Electrophoresis and western blot were performed as standard protocols describe^79,80^. Blots were developed with Westen blot developer Amersham Imager 600 (Thermo Fisher, software version 1.2.0). Uncropped and unprocessed scans of the blots can be found in the Source Data file.

Biotinylation assay to pull down cell membrane associated proteins was performed using membrane-impermeable Sulfo-NHS-Biotin. Cells were washed with ice-cold PBS, incubated with 0.25 mM membrane-impermeable EZ-Link Sulfo-NHS-Biotin on ice for 1 h (Thermo Fisher Scientific, Waltham, USA) and repetitively rinsed again in ice-cold PBS containing 100 mM Glycin. Cells lysis was done in buffer containing 50 mM NaCl, 10 mM PIPES, 3 mM MgCl_2_, 1% Triton X-100 and protease inhibitors. To detect biotinylated and thus membrane-associated proteins supernatants were collected after centrifugation and molecules were pulled down by NeutrAvidin (HighCapacity)-agarose (Thermo 3 Fisher Scientific). Precipitated molecules were suspended in 3x Laemmli buffer with 50 mM dithiothreitol (AppliChem).

The following primary antibodies were used: anti-Dsg3 rabbit polyclonal (dilution 1:1000) (Biozol, Eching, Germany), anti-GAPDH mouse monoclonal (dilution 1:1000) (Santa Cruz, Dallas, TX, USA), anti-plakoglobin mouse monoclonal (dilution 1:1000) (Progen, Heidelberg, Germany), anti-desmoplakin rabbit polyclonal (dilution 1:2000) (Abclonal, MA, USA), anti-α-Tubulin mouse monoclonal (dilution 1:1000) (Abcam, Berlin, Germany) and anti-p-plakoglobin S665 mouse monoclonal (dilution 1:10)^22^.

The following HRP-coupled secondary antibodies have been used: peroxidase-coupled-goat-anti-rabbit or –goat-anti-mouse (dilution 1:10000) (Dianova, Hamburg, Germany) was detected using the ECL-system.

### Immunostaining

For immunostaining, human keratinocytes were fixed with PFA 2% for 10 min, permeabilized with 0.1% Triton-X-100 for 5 min and treated with bovine serum albumin and normal goat serum to block unspecific antibody binding. Murine keratinocytes and human keratinocytes (for keratin stainings) were fixed using ice-cold ethanol for 30 min and subsequently ice-cold aceton for 3 min. Afterwards, cells were treated with bovine serum albumin and normal goat serum to block unspecific antibody binding. Tissues were fixed in PFA 2% for 15 min, permeabilized with 1% Triton-X-100 for 40 min and treated with bovine serum albumin in combination with normal goat serum to block unspecific antibody binding.

Subsequently, samples were incubated at 4° with the following primary antibodies: anti-desmoglein-3 rabbit polyclonal (dilution 1:100) (Biozol, Eching, Germany), anti-desmoglein-3 mouse monoclonal (dilution 1:100) and anti-occludin rabbit polyclonal (dilution 1:100) (both Invitrogen, Carlsbad, CA, USA), anti-desmoglein-1 rabbit polyclonal (dilution 1:100) and desmoplakin rabbit polyclonal (1:100) (both Abclonal, MA, USA), anti-Desmoglein-1 mouse monoclonal (dilution 1:100) (Progen, Heidelberg, Germany), anti-E-cadherin mouse monoclonal (dilution 1:100) (BD, Eysins, Switzerland), anti-β-catenin mouse monoclonal (dilution 1:100) (BD, Eysins, Switzerland), anti-panCK- FITC mouse monoclonal (dilution 1:200) (Sigma Aldrich, St. Louis, MO), anti-panCK mouse monoclonal (dilution 1:200) (Sigma Aldrich, St. Louis, MO), anti-CK14 mouse monoclonal (dilution 1:100) and anti-Loricrin rabbit polyclonal (dilution 1:200) (both Abcam, Berlin, Germany), anti-plakoglobin mouse monoclonal (dilution 1:100) (Progen, Heidelberg, Germany), anti-Dsc1 rabbit polyclonal (dilution 1:100) (Abbexa, Cambridge, UK) and anti-p-plakoglobin S665 mouse monoclonal (dilution 1:10)^22^. Specificity of Dsg3 antibodies was tested before using murine keratinocytes isolated from Dsg3 ko animals (Biozol antibody, Western blot)^75^ and human keratinocytes deficient for Dsg3 (Invitrogen antibody, immunostaining)^16^. As secondary antibodies alexa488-, Cy2- or Cy3-coupled goat-anti-rabbit or goat-anti-mouse secondary antibodies (dilution 1:600) (all dianova) were used. Phalloidin Alexa 488 (dilution 1:100) (Molecular Probes, Eugene, OR, USA) was used to stain actin. 4′,6-diamidin-2-phenylindol was added to secondary antibody incubation (DAPI, 1 mg/ml). Afterwards probes were mounted with NPG (1% n-Propylgallat and 60% Glycerin in PBS). Images were acquired with a Leica SP5 confocal microscope equipped with a 63× oil objective (Leica, Wetzlar, Germany, software version for data acquisition and analysis: 3.4.1.17822).

### HE staining

HE staining was performed according to standard procedures and mounted in DEPX (Sigma‐Aldrich).

### Stimulated emission depletion (STED) microscopy

Murine wt keratinocytes were seeded on #1.5 coverslips (VWR International). At 90 % confluence, cells were switched to high Ca^2+^ Medium for 48 h. Differentiated cells were incubated with apremilast (concentration 10 µM) for 2 h and subsequently fixed with pure ethanol for 30 min on ice followed by 3 min aceton at room temperature. Blocking was done with bovine serum albumin (BSA) NGS. Primary antibodies anti-Dp rabbit polyclonal (Abclonal, MA, USA), and anti-CK14 mouse monoclonal (Abcam, Cambridge, UK) were applied 1:100 in PBS overnight at 4 °C or for 3 h at room temperature respectively. After 3 washing steps with PBS, secondary antibodies conjugated with Alexa Fluor-594 (Abcam, Cambridge, UK) or STAR Red (Abberior, Göttingen, Germany) were incubated 1:600 in PBS at room temperature for 1 h. Afterwards the cells were mounted with ProLong Diamond Antifade Mountant (Thermo Fisher Scientific). STED microscopy images were taken with the Expert line setup from Abberior with STED Imspector software (version: 163-12585 W2040; Abberior, Göttingen, Germany). A 100 ×1.4 UPlanSApo objective was used and pixel size was set to 20 nm. To achieve the STED effect a 775 nm and 595 nm pulsed laser at 20% laser power and a gating value of 800 ps was applied. Acquired images were analyzed in the ImageJ software (NIH, Bethesda, USA).

### AFM measurements

AFM measurements on living HaCaT or murine keratinocytes were performed using a well-established protocol^52,57^. A NanoWizard® 3 AFM (JPK-Instruments, Berlin, Germany) mounted on an inverted optical microscope (Carl Zeiss, Jena, Germany) or Nanowizard 4 AFM (JPK-Instruments, Berlin, Germany) mounted on an inverted optical microscope (IX73 Olympus, Hamburg, Germany), which both allow selection of the scanning area by visualizing the cells with a 63x objective were used. JPK NanoWizard software v.6, version 6.1.176 was used for data acquisition (JPK Instruments, Berlin, Germany). Pyramidal-shaped D-Tips of Si_3_N_4_ MLCT cantilevers (Bruker, Mannheim, Germany) with a nominal spring constant of 0.03 N/m and tip radius of 20 nm were functionalized with purified Dsg3-Fc or Dsg1-Fc (concentration of 0.15 mg/ml) as described before^81^. Here, a flexible heterobifunctional acetal-polyethylenglycol (PEG, BroadPharm, San Diego, US) was interspaced between the tip and the recombinant Dsg-Fc. AFM was used either in Quantitative Imaging (QI, for overview images) or Force Mapping (FM, for force measurements)-mode. In QI mode an area of 20 × 30 µm was sampled with setpoint of 0.3 nN and a pulling speed of 50 µm/s; a Z-length of 2 µm and an additional retract of 150 nm. These settings were applied to avoid cell alterations due to mechanical stress and allow to identify areas of cell-cell-contact further referred as cell borders. In FM mode each map consists of distinct force-distance cycles with each cycle representing one pixel of the map covering an area of 1.25 × 5 µm along a cell border. Settings were adapted to setpoint 0.5 nN, pulling speed 10 µm/s, Z-length 2 µm and a resting contact time 0.1 s.

### Fluorescence recovery after photobleaching (FRAP)

For FRAP experiments wt and Pg-S665A murine keratinocytes were transiently transfected with pEGFP-C1-Dsg3 (kindly provided by Dr. Yasushi Hanakawa, Ehime University School of Medicine, Japan) using Lipofectamine 3000 (Invitrogen, Carlsbad, USA)^82,83^. Cells were switched to high Ca^2+^ (1.2 mM) 24 h after transfection and treated with vehicle or apremilast 48 h after Ca^2+^ switch for 2 h. FRAP experiments were conducted on an SP5 inverted microscope with a x63 HC PL APO NA = 1.2 objective (Leica, Wetzlar, Germany) in a cage incubator (OKOLAB, Burlingame, CA) at 37 °C at constant humidity with 5 % CO_2_ using the FRAP wizard software (Leica). Region of Interest (ROI) for bleaching was chosen along the membrane of two transfected adjacent cells. Dsg3 signal of a ROI was bleached using an Argon laser ($\lambda=488\,{{{{{\rm{nm}}}}}}$) at 100% transmission and recovery of the fluorescence was recorded for 180 s until a stable fluorescence intensity was reached. Immobile fraction and recovery halftime (τ_1/2_) was determined to investigate Dsg3 mobility.

### Image processing and statistical analysis

Origin Pro 2016G 64 bit (OriginLab, Northhampton, MA, USA) was used for analyzing cAMP Assay and AFM data. Image processing was performed with Photoshop CS5 (Adobe, San José, USA) and LAS X life science (Leica, Wetzlar, Germany). Image J (National Institutes of Health) was used to quantify fluorescence intensity (fluorescence stainings), band intensity (Western blot analysis) and blister length (HE staining). AFM data were analyzed with JPK Data Processing software (version 6.1.163, JPK-Instruments, Berlin, Germany). For statistics and creating graphs GraphPad Prism (GraphPad Software, version: 9.4.1., San Diego, CA, USA) was used. To compare means of more than 2 samples to respective controls one-way ANOVA with Bonferroni correction was used and significance was determined for a *p*-value of <0.5. For two independent groups unpaired T-test was applied.

### Reporting summary

Further information on research design is available in the Nature Portfolio Reporting Summary linked to this article.

## Supplementary information

Supplementary material

Reporting Summary

## Data Availability

All data supporting the findings of this study are available within the main manuscript and the supplementary files. A reporting summary for this article is available as a Supplementary File. Source data are provided with this paper.

## Source data

Source Data
