# Author Correction: Apremilast prevents blistering in human epidermis and stabilizes keratinocyte adhesion in pemphigus

**DOI:** 10.1038/s41467-023-36464-6

**Published:** 2023-02-07

**Authors:** Anna M. Sigmund, Markus Winkler, Sophia Engelmayer, Daniela Kugelmann, Desalegn T. Egu, Letyfee S. Steinert, Michael Fuchs, Matthias Hiermaier, Mariya Y. Radeva, Franziska C. Bayerbach, Elisabeth Butz, Stefan Kotschi, Christoph Hudemann, Michael Hertl, Sunil Yeruva, Enno Schmidt, Amir S. Yazdi, Kamran Ghoreschi, Franziska Vielmuth, Jens Waschke

**Affiliations:** 1 Chair of Vegetative Anatomy, Institute of Anatomy, Faculty of Medicine, LMU Munich, Munich, Germany; 2 Department of Dermatology and Allergology, Philipps-University Marburg, Marburg, Germany; 3 Lübeck Institute of Experimental Dermatology (LIED), University of Lübeck, Lübeck, Germany; 4 Center for Research on Inflammation of the Skin, University of Lübeck, Lübeck, Germany; 5 Department of Dermatology, Rheinisch-Westfälische Technische Hochschule Aachen (RWTH) Aachen University, Aachen, Germany; 6 Department of Dermatology, Eberhard Karl University of Tuebingen, Tuebingen, Germany; 7 Department of Dermatology, Venereology and Allergology, Charité-Universitätsmedizin Berlin, Berlin, Germany

**Keywords:** Skin diseases, Mechanisms of disease, Desmosomes

Correction to: *Nature Communications* 10.1038/s41467-022-35741-0, published online 09 January 2023

This Article contains an error in the spelling of the author Matthias Hiermaier, which is incorrectly given as Matthias Hiermair. The error has been corrected in the PDF or HTML versions of the Article.

