# Supplementary material for: Apremilast prevents blistering in human epidermis and stabilizes keratinocyte adhesion in pemphigus
Source: Nat Commun. 2023 Jan 9;14:116. doi: 10.1038/s41467-022-35741-0 (PMC9829900; doi:10.1038/s41467-022-35741-0)
Supplement: Supplementary file 1 — Supplementary material [file 41467_2022_35741_MOESM1_ESM.pdf]

Supplementary Information

Supplementary Tables

Supplementary Table 1: List of used primer

Purpose/Aim	Primer	Sequence 5'-> 3'
To generate the short arm (SA)	I143.1	CAGGATATCGCCCCTAATACCTGAGC
To generate the short arm (SA)	I143.2	CAAGCGGCCGCCTCCTGACTGCTGAG AATAC
To generate the long arm (LA)	I143.8	CTAGGCGCGCCGGACTCTTGGCTGTA GTAG
To test the presence of mutation in ES cells<br>To generate the long arm (LA)<br>Overlay-extension PCR	I143.4	GTTCCTGCAGGGTGTCTGCTCAGGTAT TAGG
To test the presence of mutation in ES cells. Overlay-extension PCR	I143.5	CAAGGCGCGCCAGAGGTCCTGAGTTC AAGTC
Generation of probe for Southern Blot	I143.21	AGGCTGTCGAGATGATTC
Generation of probe for Southern Blot	I143.22	CAGAGATCAGCCTGTCTATG
Overlay-extension PCR	I143.6	GAATTGGTGAGCTCCACAGCCACTCG CTTACGGTAATC
Overlay-extension PCR	I143.7	GATTACCGTAAGCGAGTGGCTGTGGA GCTCACCAATTC
Screening for Neo resistance cassette deletion	I143.27	GACACATGCGAACATACC
Screening for Neo resistance cassette deletion	I143.28	CCTGTCTAGCCATCTTAGG
Screening for Neo resistance cassette	Neo.MP1	GCTGTGCTCCACGTTGTCAC
Screening for Neo resistance cassette	Neo.MP5	GGAAAGCTGGGCTTGCATCTC
Screening for Neo resistance cassette	Neo.MP6	GGAGCGGCGATACCGTAAAG
Screening for Flp recombinase allele	SD24	CTAATGTTGTGGGAAATTGGAGC
Screening for Flp recombinase allele	SD25	CTCGAGGATAACTTGTTTATTGC
Detection of the S665A mutation in mice	I143.25	CTAGCTCCTGTACTCCTCTG
Detection of the S665A mutation in mice	I143.26	TGGTGGCTCATGACTCTC
Screening PCR	I143.9	GTAACCCAGGCTGTCCTTAG
Screening PCR	5182LRP CR2	GTTGTGCCCAGTCATAGCCGAATAG
presence of Flp recombinase allele	SD24	CTAATGTTGTGGGAAATTGGAGC
presence of Flp recombinase allele	SD25	CTCGAGGATAACTTGTTTATTGC

Supplementary Table 2: Body donor information

body donor	age	sex
01	81	male
02	83	female
03	86	female
04	97	female
05	80	male
06	80	female
07	91	female
08	74	male
09	83	female

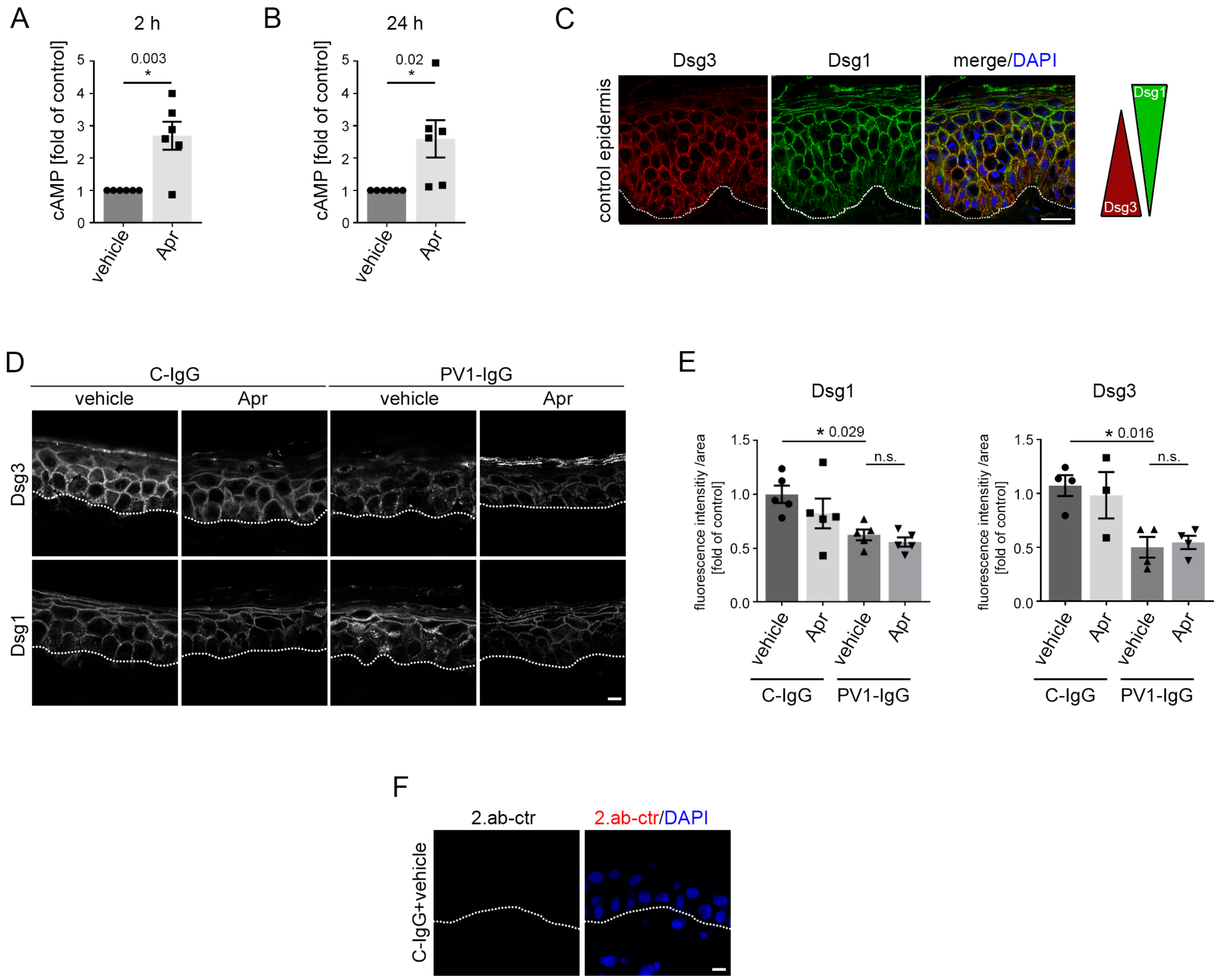

Figure S1: ELISA for cAMP, Dsg immunostaining of *ex vivo* skin model.

cAMP ELISA of HaCaT cell lysates after incubation with apremilast or vehicle (DMSO) for 2 h (A) or 24 h (B) revealed a significant increase of cAMP levels after apremilast treatment under both conditions (n=6). (C) Staining of Dsg1 and Dsg3 in human epidermis revealing a inverse staining pattern. Scale bar = 25 µm. Representative of n=4. (D) Immunostaining against Dsg1 and Dsg3 in *ex vivo* skin. Apremilast did not improve PV1-IgG-induced Dsg depletion. Scale bar = 7.5 µm. Representative of n=3-5. (E) Quantification of *ex vivo* model. PV-IgG significantly reduced Dsg1 (n=5) and Dsg3 (n=4; n=3 for C-IgG+Apr) fluorescence intensity which was not rescued by apremilast treatment. (F) Secondary antibody control of immunostaining of *ex vivo* skin samples. Representative of n=4. White dotted line marks basement membrane. Scale bar = 7.5 µm. Columns indicate mean value normalized to control ± SEM. *P<0.05 (exact values are depicted in the figure). Two-tailed Student's t-test (B, C), One-way ANOVA with Bonferroni correction (E). Source data are provided as a Source Data file.

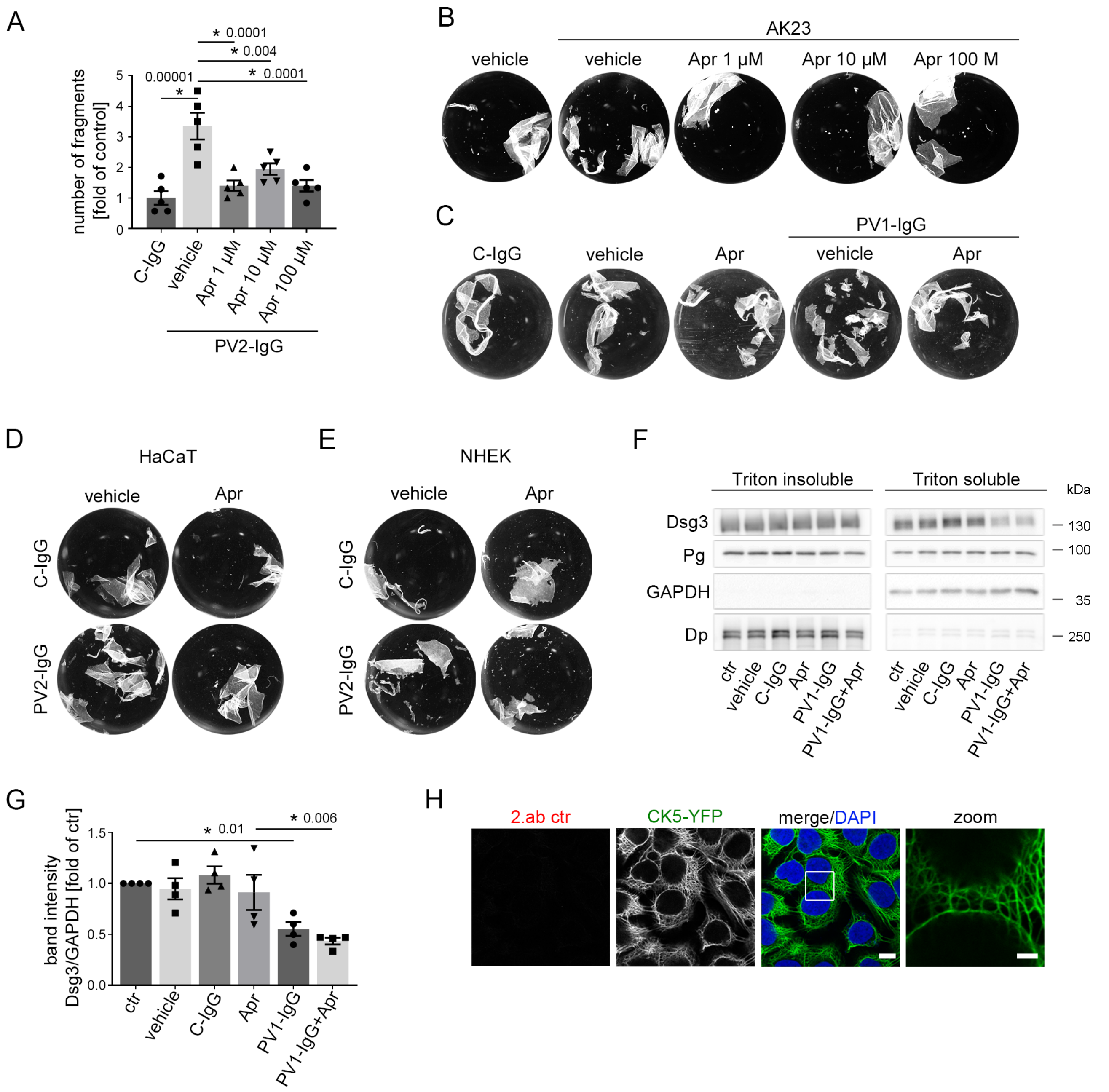

Figure S2: Images of dissociation assays and control IF and apremilast rescues PV-IgG-induced loss of intercellular adhesion but not Dsg3 depletion.

(A) Dispase-based keratinocyte dissociation assay of human keratinocytes. 1 h pre-incubation of all indicated concentrations of apremilast attenuated PV2-IgG loss of cell cohesion in HaCaT cells (n=5). Representative images to dissociations assays in Fig. 2A (B), 2B (C), 2C (D), 2D (E), respectively. (F) Representative Western blot of HaCaT Triton-soluble and Triton-insoluble, i.e. desmosome containing fractions showed no effect of apremilast on PV1-IgG-induced Dsg3 depletion. Desmoplakin (Dp) and Glycerol-3-phosphate dehydrogenase (GAPDH) served as loading controls for the respective fraction. (G) Quantification of Triton-soluble fraction of Western blots (n=4). (H) Secondary antibody control for immunostaining in CK5-YFP-HaCaT. Scale bar = 10 µm. Zoom in areas marked with white rectangles. Scale bar (zoom) = 2.5 µm. Representative of n=4. Columns indicate mean values normalized to control ± SEM. *P<0.05 (exact values are depicted in the figure). One-way ANOVA with Bonferroni correction. Plakoglobin (Pg). Source data are provided as a Source Data file.

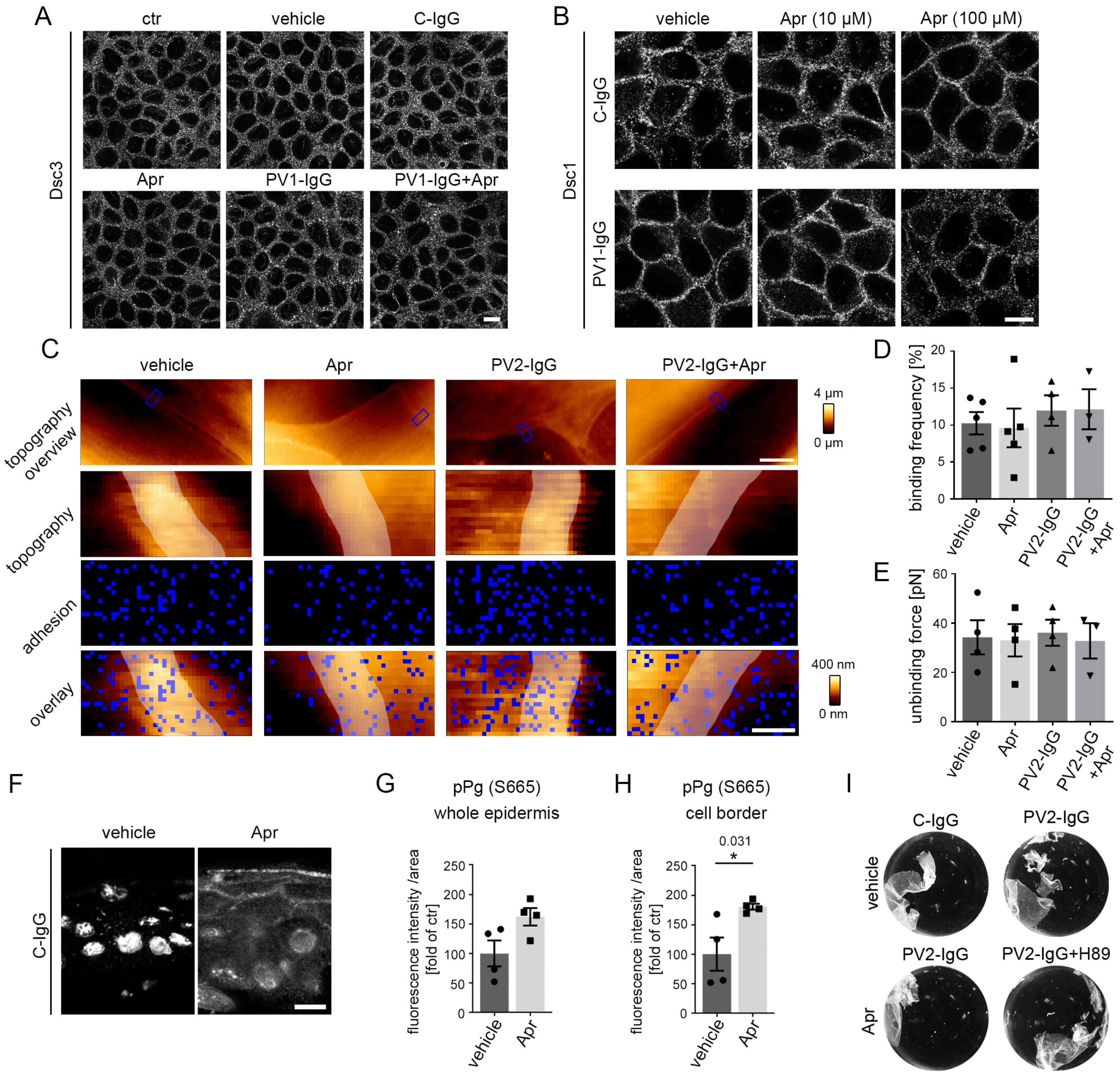

Figure S3 Apremilast had no effect on Dsc stainings and Dsg1 binding properties but did induce localisation of phosphorylated Pg at the cell border in a human *ex vivo* skin model.

(A) PV1-IgG did not change Dsc3 staining in HaCaT keratinocytes. Apremilast (Apr, 100 µM) had no impact on Dsc3 staining in controls or PV-IgG-treated cells (n=4). Scale = 10 µM. (B) PV1-IgG did not change Dsc1 staining in HaCaT keratinocytes. Apremilast (10 µM and 100 µM) had no impact on Dsc1 staining under control or PV-IgG treated cells (n=4). Scale = 10 µM. (C) Topography overview images of atomic force microscopy measurements (AFM) on living murine keratinocytes treated with PV2-IgG and apremilast using a Dsg1-functionalized tip revealing cell borders bridged by dense filamental structures. Scale bar = 10 µm. Small areas along the cell borders (blue rectangles) were chosen for adhesion measurements. In these areas each blue pixel represents a force-distance-curve/Dsg1-specific binding event. Cell borders are highlighted in light grey. Scale bar = 1 µm. (D-E) Quantification of AFM adhesion measurements (D: vehicle, Apr: n=5, PV1-IgG: n=4, PV1-IgG+Apr: n=3; E: n=4, PV1-IgG+Apr: n=3).(F) Immunostaining against phosphorylated Pg of *ex vivo* human skin showed weak dotted staining of the membrane and strong staining of the nuclei. After injection with 100 µM apremilast phosphorylated Pg was clearly visable at cell borders (n=4). Scale bar = 7.5 µm. (G, H) Quantification showed an increase of pPg (S665) after apremilast injection which was significant at cell borders (H). (I) Representative pictures of dispase assay of Figure 4G. Columns indicate mean values normalized to control ± SEM. *P<0.05 (exact values are depicted in the figure). Two tailed Student‘s t-test (G, H), One-way ANOVA with Bonferroni correction (D, E). Source data are provided as a Source Data file.

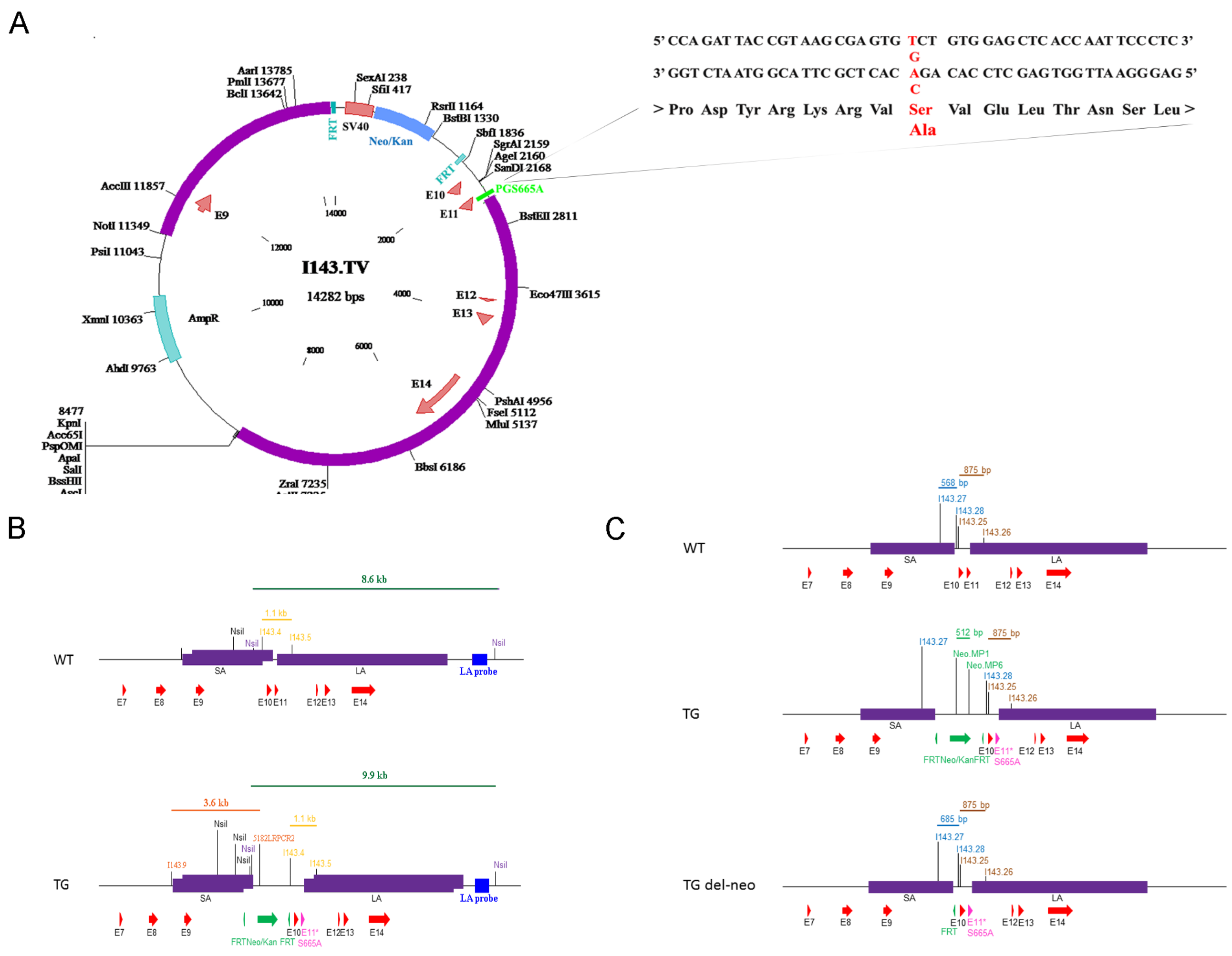

Figure S4: Generation of Knock-in mouse model for Jup S665A mutation.

(A) The mutated Jup allele. The 1993 T>G mutation in exon 11 results in the exchange of serine (Ser) for alanine (Ala, red). (B) Schematic drawing of the different Jup alleles. The upper panel shows the wild type (WT) allele, the lower panel shows the targeted (TG) allele. Homologous regions (SA: short arm of homology; LA: long arm of homology) used for recombination are depicted as purple boxes. The mutation in exon 11 (E11* S665A, pink) is inserted via homologous recombination together with a FRT-flanked neomycin cassette (green). The primers I143.9 and 5182LRPCR2 were used for PCR screening of recombined ES cells. The corresponding fragment size is indicated in orange. Primers used for confirmation of the S665A mutation are indicated in yellow. The restriction enzyme NsiI, used for Southern blot analysis, and the corresponding 3' external probe (LA probe) and fragments are indicated in blue. (C) Schematic drawing of the WT and the targeted locus of plakoglobin, with (TG) and without the neomycin resistance cassette (TG del-neo). The primers used for genotyping and the corresponding amplicon sizes (blue: del-neo PCR, brown: PCR for confirmation of the S665A mutation, green: neomycin PCR) are mapped on the different loci.

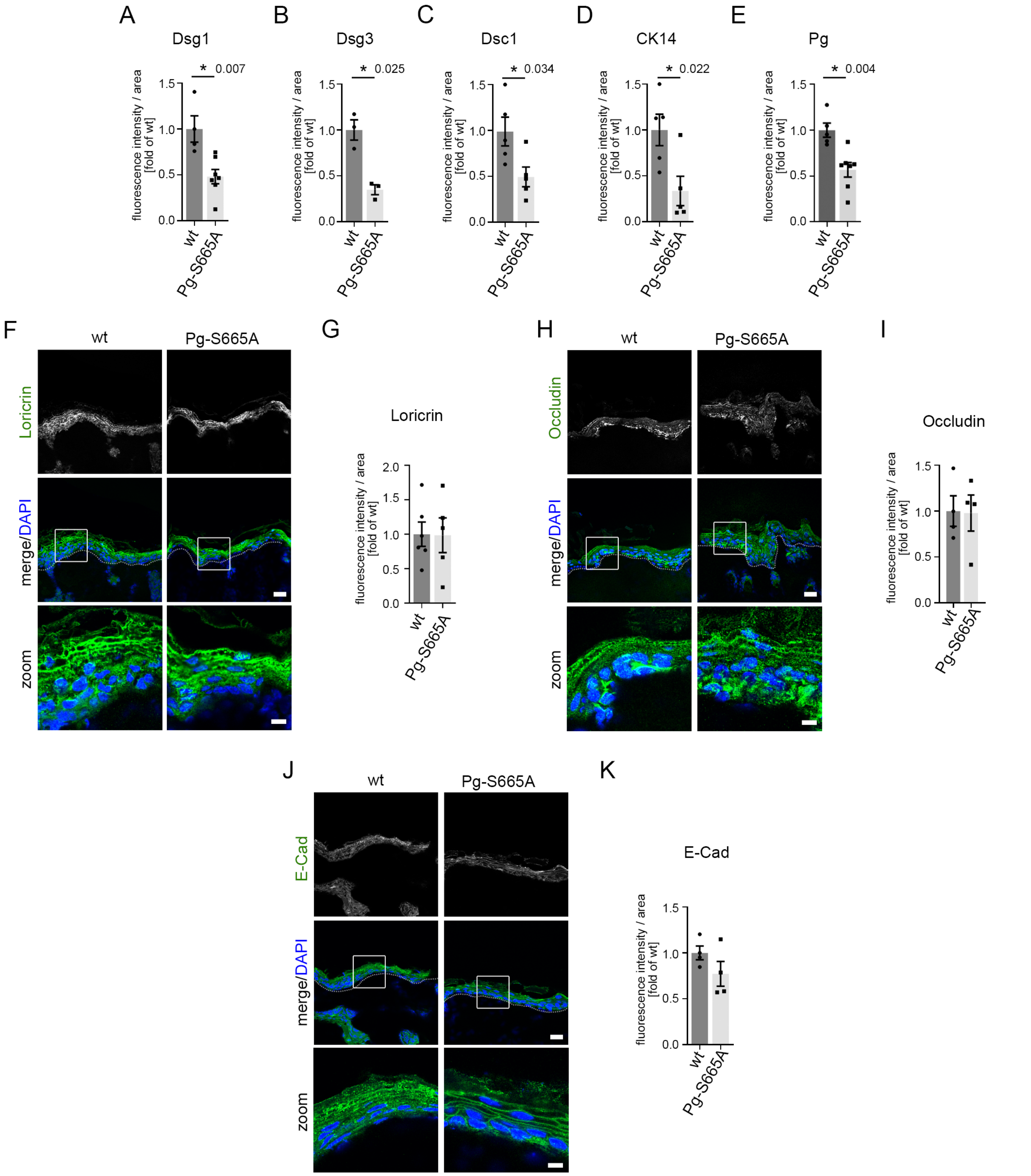

Figure S5: Protein expression pattern in murine epidermis.

(A-E) Quantification of fluorescence intensity of the respective protein from Fig. 5 (n≥3, exact values are depicted in Fig.5). (F) The differentiation marker loricrin was not changed in Pg-S665A epidermis. Representative of n=5-6. (G) Quantification of loricrin in murine epidermis (wt: n=6, Pg-S665A: n=5). (H) The tight junction marker occludin was not altered in Pg-S665A epidermis. Representative of n=4. (I) Quantification of occludin in murine epidermis (n=4). (J) E-cadherin (E-Cad) was not altered in Pg-S665A epidermis. Representative of n=4. (K) Quantification of E-Cad in murine epidermis (n=4). White dotted lanes represent basement membranes. Scale bar = 25 µm. White rectangles represent areas for zoom in. Scale bar (zoom) = 8 µm. Columns indicate mean ± SEM. *P<0.05 (exact values are depicted in the figure). Two-tailed Student's t-test. Source data are provided as a Source Data file.

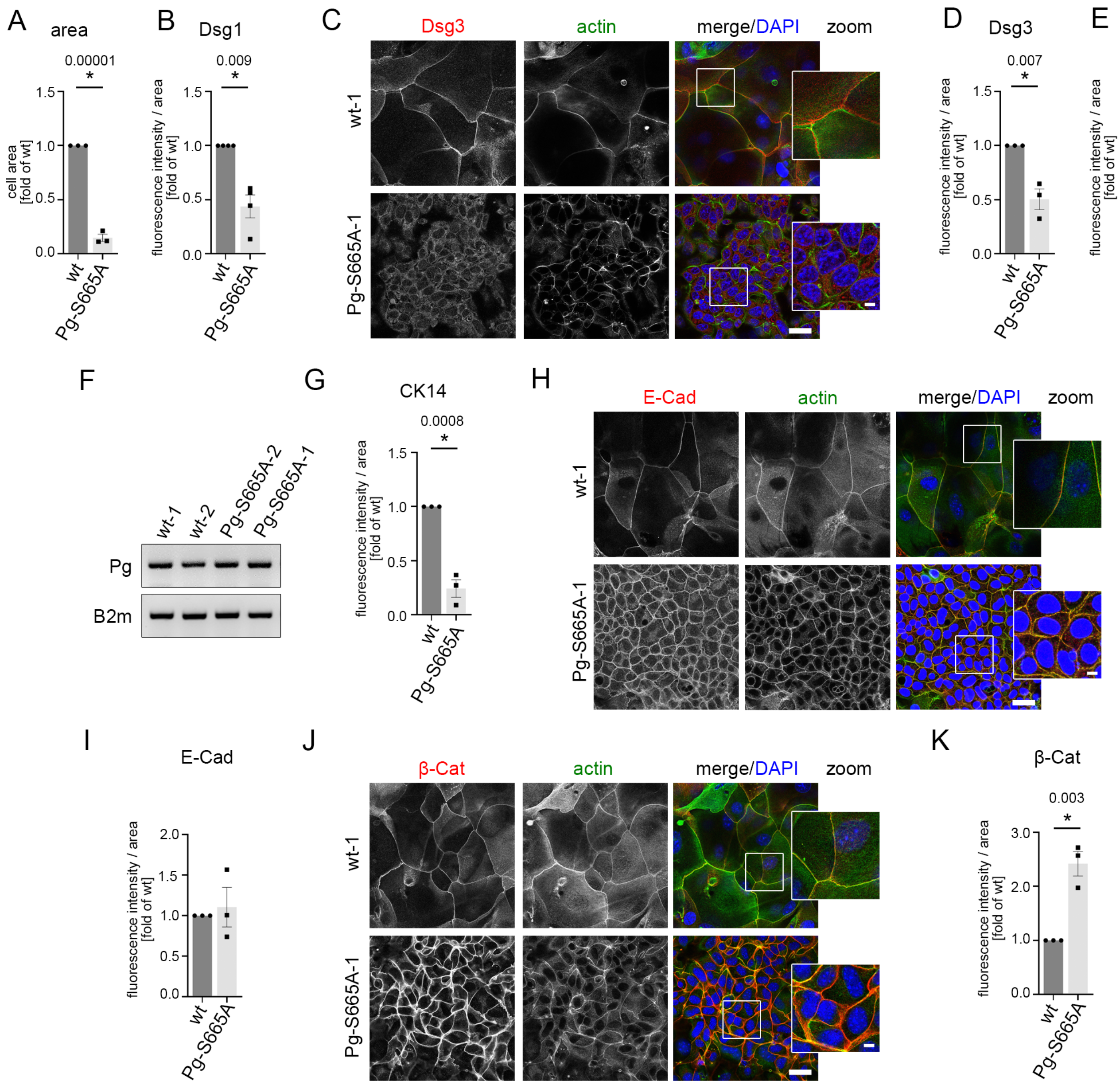

Figure S6: Keratinocytes phospho-deficient at Pg S665 show alterations in desmosomal protein distribution and compensatory effect on adherens junction protein.

(A) Quantification of cell size (area) of wt and Pg deficient keratinocytes (n=3). (B, E, G) Quantification of immunostainings of Dsg1 (n=4), Pg and Ck14 (n=3) in Fig 6. (C) Co-staining of Dsg3 and actin and quantification of Dsg3 staining (D) revealed impaired and in part fragmented distribution of Dsg3 along the cell borders in Pg-S665A keratinocytes (n=3). (F) mRNA of Pg is unaltered in Pg-S665A keratinocytes compared to wt. Beta-2-microglobulin (B2m) was used as input control. Representative of n=4 and 3 for Pg-S665A-2. (H) Co-staining of E-cadherin (E-Cad) and actin and quantification of E-Cad staining (I) showed no difference in Pg-S665A keratinocytes (n=3). (J) Co-staining of β-catenin and actin and quantification (K) demonstrated a clear upregulation of β-catenin along cell borders in Pg-S665A keratinocytes (n=3). Scale bar = 25 µm. White rectangles depict areas chosen for zoom ins. Scale bar (zoom) = 5 µm. Columns indicate mean value ± SEM. P<0.05 (exact values are depicted in the figure). Two-tailed Student's t-test. Source data are provided as a Source Data file.

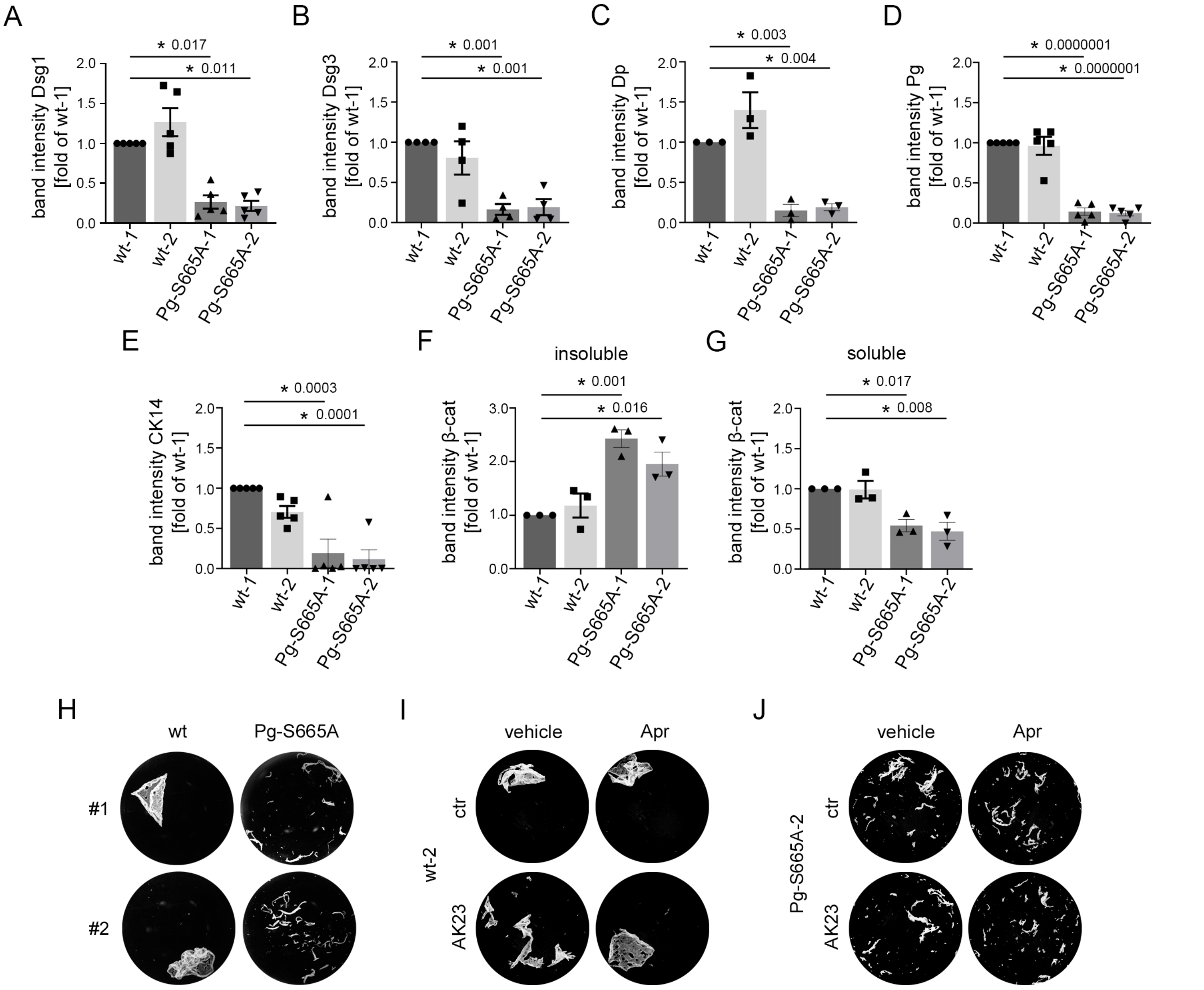

Figure S7: Keratinocytes phospho-deficient at Pg S665 show alterations of desmosomal proteins and disturbed cell adhesion.

(A-G) Quantification of Western blot in Fig 6F of the triton insoluble fractions for Dsg1 (A, n=5), Dsg3 (B, n=4), Dp (C, n=3), Pg (D, n=5), CK14 (E, n=5), β-Cat (F, n=3) and the soluble fraction of β-Cat (G, n=3). (H-J) Pictures of dissociation assays in wt and Pg-S665A keratinocytes from Fig. 6. N values are depicted in Fig.6. Columns indicate mean value ± SEM. P<0.05. One-way ANOVA with Bonferroni correction.

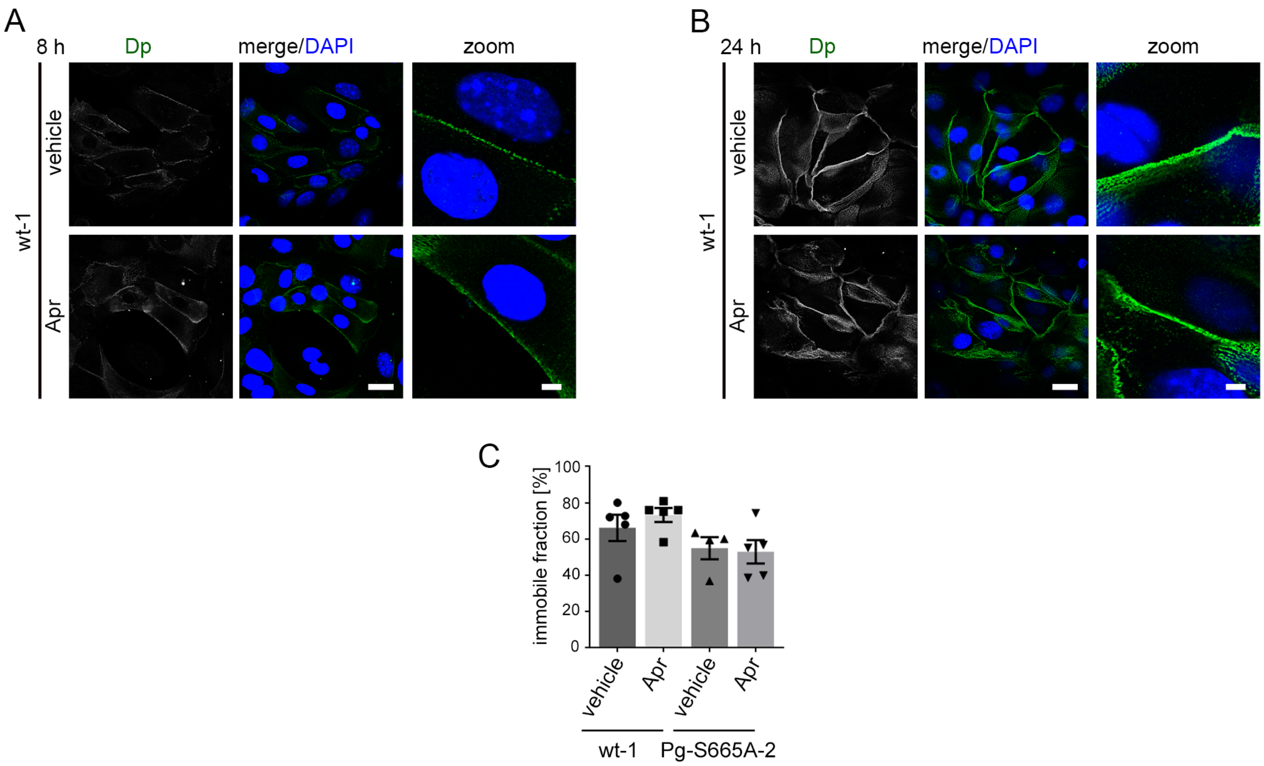

Figure S8: Apremilast has no effect on desmosome assembly 8 h and 24 h after Ca^{2+} switch and Dsg3 mobility.

(A, B) Ca^{2+} switch experiments to investigate desmosome assembly in wt murine keratinocytes. Assembly was characterized at 8 h (A) and 24 h (B) respectively. Apremilast (10 µM) did not change membrane staining of desmoplakin (Dp) after 8 h or 24 h of Ca^{2+} application, respectively. Representative of n=4. Scale= 25 µm, scale (Zoom) =5 µm. (C) Quantification of Dsg3-GFP in FRAP experiments in wt and Pg-S665A keratinocytes. Apremilast (10 µM) did not change immobile fraction of Dsg3 (n = 5, for Pg-S665A vehicle: n=4). Columns indicate mean values +/- SEM. *P<0.05. One-way ANOVA with Bonferroni correction. Source data are provided as a Source Data file.
