# Supplementary material for: Apremilast prevents blistering in human epidermis and stabilizes keratinocyte adhesion in pemphigus
Source: Nat Commun. 2023 Jan 9;14:116. doi: 10.1038/s41467-022-35741-0 (PMC9829900; doi:10.1038/s41467-022-35741-0)
Supplement: Supplementary file 2 — Reporting Summary [file 41467_2022_35741_MOESM2_ESM.pdf]

nature portfolio

Corresponding author(s): Jens Waschke and Franziska Vielmuth

Last updated by author(s): Dec 14, 2022

Reporting Summary

Nature Portfolio wishes to improve the reproducibility of the work that we publish. This form provides structure for consistency and transparency in reporting. For further information on Nature Portfolio policies, see our Editorial Policies and the Editorial Policy Checklist.

Statistics

For all statistical analyses, confirm that the following items are present in the figure legend, table legend, main text, or Methods section.

n/a	Confirmed	
☐	☒	The exact sample size (*n*) for each experimental group/condition, given as a discrete number and unit of measurement
☒	☐	A statement on whether measurements were taken from distinct samples or whether the same sample was measured repeatedly
☐	☒	The statistical test(s) used AND whether they are one- or two-sided<br>*Only common tests should be described solely by name; describe more complex techniques in the Methods section.*
☒	☐	A description of all covariates tested
☐	☒	A description of any assumptions or corrections, such as tests of normality and adjustment for multiple comparisons
☐	☒	A full description of the statistical parameters including central tendency (e.g. means) or other basic estimates (e.g. regression coefficient) AND variation (e.g. standard deviation) or associated estimates of uncertainty (e.g. confidence intervals)
☐	☒	For null hypothesis testing, the test statistic (e.g. *F*, *t*, *r*) with confidence intervals, effect sizes, degrees of freedom and *P* value noted<br>*Give P values as exact values whenever suitable.*
☒	☐	For Bayesian analysis, information on the choice of priors and Markov chain Monte Carlo settings
☒	☐	For hierarchical and complex designs, identification of the appropriate level for tests and full reporting of outcomes
☐	☒	Estimates of effect sizes (e.g. Cohen's *d*, Pearson's *r*), indicating how they were calculated

Our web collection on statistics for biologists contains articles on many of the points above.

Software and code

Policy information about availability of computer code

Data collection	jpk nano wizard software v.6, version 6.1.176 (AFM measurements), LRSX-Leica, version 3.4.1.17822 (confocal images, including FRAP wizard for FRAP experiments), Libra 120 transmission electron microscopy software, version V01.05.00.00 (electron microscopy), STED Imspector 163-12585 W2040 (STED images). WB developer Amersham Imager 600, software version 1.2.0 (Western blots), Tecan Magellan software version V7.2 (cAMP ELISA; BCA-Protein-Assay)
Data analysis	image j (NIH), jpk data processing software (version 6.1.163) , LRSX-Leica (3.4.1.17822), Adobe photoshop (version 12.0 x64), graph pad prism (version: 9.4.1), OriginPro 2016G 64bit

For manuscripts utilizing custom algorithms or software that are central to the research but not yet described in published literature, software must be made available to editors and reviewers. We strongly encourage code deposition in a community repository (e.g. GitHub). See the Nature Portfolio guidelines for submitting code & software for further information.

Data

Policy information about availability of data

All manuscripts must include a data availability statement. This statement should provide the following information, where applicable:

- Accession codes, unique identifiers, or web links for publicly available datasets
- A description of any restrictions on data availability
- For clinical datasets or third party data, please ensure that the statement adheres to our policy

The data that support the findings of this study are available in the main oder suppl. figures and in the Source data file.

Human research participants

Policy information about studies involving human research participants and Sex and Gender in Research.

Reporting on sex and gender	ex vivo pemphigus skin model: Skin was taken from cadavers of the body donor program of anatomy department of LMU Munich. Skin of male and female donors behave similar in terms of epidermal blistering when treated with pemphigus vulgaris autoantibodies. Thus, we did not perform sex- and gender-based analysis of the skin samples. Sex and age of the body donors are included in Suppl. Table 2.
Population characteristics	n/a
Recruitment	After their death, body donors voluntarily donate their bodies to the Anatomical Institute for scientific and teaching purposes. To assure viability of the tissue we take skin samples only from donors deceased less then 24h before sample acquisition and perform viability assays. No further selection of the cadavers is made by the researchers.
Ethics oversight	Experiments are under the allowance of the body donor program of the LMU. Donors gave their written and informed consent for the usage of skin samples after they deceased. The local ethical committee confirmed that no further ethical approvement is needed for the experimental protocol.

Note that full information on the approval of the study protocol must also be provided in the manuscript.

Field-specific reporting

Please select the one below that is the best fit for your research. If you are not sure, read the appropriate sections before making your selection.

☒ Life sciences ☐ Behavioural & social sciences ☐ Ecological, evolutionary & environmental sciences

For a reference copy of the document with all sections, see nature.com/documents/nr-reporting-summary-flat.pdf

Life sciences study design

All studies must disclose on these points even when the disclosure is negative.

Sample size	For animal studies power analysis was performed to determine number of animals. For in vitro and ex vivo experiments no calculation was performed. Individual experiments were repeated multiple times as indicated to ensure robustness and reproducibility.
Data exclusions	Dissociation assays: Datasets in which control monolayers reveal a >3fold fragmentation compared to average were excluded. In vivo pemphigus mouse model: Animals were monitored regarding their individual stress following guidelines of the german Tierschutzgesetz using a score sheet for the respective experimental setup confirmed by the Regierung von Oberbayern (number: Vet 02_21_205). Animals showing characteristics of high stress levels were excluded and sacrified. Immunostaining: Data were excluded if secondary antibody control revealed unspecific staining.
Replication	All experiments were repeated independently multiple times. Number of successfull replicates of distinct samples/animals is given in the respective figure legend. This number indicated how many independent experiments were performed.
Randomization	Cell culture experiments (immunostaining, STED, Western blot, dissociation assay,AFM, cAMP ELISA): all conditions to be compared were performed on the same multi well plate in which the cells were seeded to provide comparable growing conditions. Respective conditions were randomly distributed along the wells used and changed between the experiments. For dissociation assays lids with respective lettering was removed and counting of fragments was performed blinded (see paragraph below). Animal experiments: animals were subdivided in experimental groups with each group receiving a certain treatment from an independent researcher to exclude preselection from the experimentators. Injection and application of mechanical stress was performed by different researcher and animals were blinded using a numeric system (see paragraph below).<br>No randomization was possible due to technical limitations (e.g. requirement to select transfected cells in FRAP experiments or suitable cells (with no debris) for single molecule force measurements with AFM technique) or limitation of the samples (in the human ex vivo model all conditions for 1 independent replicate are applied on the skin samples of the same body donor).

For all further methods no randomization is applicable (generation of mouse model, purification of IgG fractions or proteins, cell culture).

Blinding

For dissociation assays lids with respective lettering was removed and counting of fragments was performed blinded.
Injection and application of mechanical stress was performed by different researcher and animals were blinded using a numeric system.
Data analysis (AFM; electron microscopy) was performed blinded using a numeric system.
No blinding was applicable for immunostaining, cAMP ELISA and Western blot experiments. However, the experiment and analysis was performed by independent researcher to avoid biased analysis.
For FRAP, AFM and STED experiments no blinding was applicable due to technical limitations (e.g. application of mediators during experiments with repeated measurements).
For all further methods no blinding is applicable (generation of mouse model, purification of IgG fractions or proteins, cell culture).

Reporting for specific materials, systems and methods

We require information from authors about some types of materials, experimental systems and methods used in many studies. Here, indicate whether each material, system or method listed is relevant to your study. If you are not sure if a list item applies to your research, read the appropriate section before selecting a response.

Materials & experimental systems

n/a	Involved in the study
☐	☒ Antibodies
☐	☒ Eukaryotic cell lines
☒	☐ Palaeontology and archaeology
☐	☒ Animals and other organisms
☒	☐ Clinical data
☒	☐ Dual use research of concern

Methods

n/a	Involved in the study
☒	☐ ChIP-seq
☒	☐ Flow cytometry
☒	☐ MRI-based neuroimaging

Antibodies

Antibodies used

anti-Dsg3 rabbit polyclonal (Biozol, Eching, Germany), catalog number: 62720-120, no clone name provided as antibody is polyclonal.
anti-GAPDH mouse monoclonal (Santa Cruz, Dallas, TX, USA): catalog number: sc-47724, clone number: 0411.
anti-plakoglobin mouse monoclonal (Progen, Heidelberg, Germany), catalog number: 61005, clone: PG 5.1
anti-desmoplakin rabbit polyclonal (Abclonal, MA, USA), catalog number: A7635, no clone name provided as antibody is polyclonal.
anti-α-Tubulin mouse monoclonal (Abcam, Berlin, Germany), catalog number: ab7291, clone: [DM1A]
anti-p-plakoglobin S665: mouse monoclonal, self-made
peroxidase-coupled-goat-anti-rabbit (Dianova, Hamburg, Germany): catalog number: 11-035-003, no clone provided as antibody is polyclonal.
peroxidase-coupled –goat-anti-mouse (Dianova, Hamburg, Germany): catalog number: 115-035-068, no clone provided as antibody is polyclonal.
anti-desmoglein-3 mouse monoclonal Invitrogen, Carlsbad, CA, USA: catalog number: MA5-16025, clone number: 5G11).
anti-occludin rabbit polyclonal (Invitrogen, Carlsbad, CA, USA): catalog number: 40-4700, clone number: no clone name provided as antibody is polyclonal.).
anti-desmoglein-1 rabbit polyclonal (Abclonal, MA, USA): catalog number: A9812, clone number: no clone name provided as antibody is polyclonal.).
anti-Desmoglein-1 mouse monoclonal (Progen, Heidelberg, Germany, catalog number: 651111, clone number: Dsg1-P124).
anti-E-cadherin mouse monoclonal (BD, Eysins, Switzerland, catalog number: 610181, clone number: 36/E-Cadherin).
anti-β-catenin mouse monoclonal (BD, Eysins, Switzerland, catalog number: 610154, clone number: 42/96).
anti-panCK- FITC mouse monoclonal (Sigma Aldrich, St. Louis, MO, catalog number: F3418, clone number: clone C-11).
anti-panCK mouse monoclonal (Sigma Aldrich, St. Louis, MO, catalog number: C2931, clone number: clone C-11).
anti-CK14 mouse monoclonal (Abcam, Berlin, Germany, catalog number: ab7800, clone number: LL002).
anti-Loricrin rabbit polyclonal (Abcam, Berlin, Germany): catalog number: ab85679, no clone available as antibody is polyclonal.
anti-Dsc1 rabbit polyclonal (Abbexa, Cambridge, UK): catalog number: abx176152, no clone available as antibody is polyclonal.
anti-Dsc3 rabbit polyclonal (LSBio, Seatlle, USA): catalog number: LS-B9474-200, no clone available as antibody is polyclonal.
secondary antibodies alexa488-goat-anti-mouse, (Dianova, Hamburg, Germany), catalog number: 115-547-003, no clone provided as antibody is polyclonal.
Cy2-coupled goat-anti-rabbit secondary antibodies (Dianova, Hamburg, Germany), catalog number: 111-225-144, no clone provided as antibody is polyclonal.
Cy3-coupled goat-anti-rabbit secondary antibodies (Dianova, Hamburg, Germany), catalog number: 111-165-003, no clone provided as antibody is polyclonal.
Cy2-coupled goat-anti-mouse secondary antibodies (Dianova, Hamburg, Germany): catalog number: 115-225-075, no clone provided as antibody is polyclonal.
Cy3-coupled goat-anti-mouse secondary antibodies (Dianova, Hamburg, Germany), catalog number: 115-165-164, no clone provided as antibody is polyclonal.

Validation

anti-Dsg3 rabbit polyclonal (Biozol, Eching, Germany): Specificity was tested in our laboratory in murine keratinocytes isolated from Dsg3 KO animals (Sigmund et al, Front Immunol, 2020; Biozol antibody, Western blot).

All further antibodies were tested for their specificity and species reactivity by manufacturers. Please find enclosed the respective information:

anti-GAPDH mouse monoclonal (Santa Cruz, Dallas, TX, USA): validation statement and references can be found on manufacturers homepage: https://datasheets.scbt.com/sc-47724.pdf "GAPDH (0411) is recommended for detection of GAPDH of human origin by Western Blotting (starting dilution 1:200, dilution range 1:100-1:1000), immunoprecipitation [1-2 µg per 100-500 µg of total protein (1 ml of cell lysate)], immunofluorescence (starting dilution 1:50, dilution range 1:50-1:500) and immunohistochemistry (including paraffin-embedded sections) (starting dilution 1:50, dilution range 1:50-1:500); not recommended for detection of GAPDH of mouse or rat origin."; relevant references: 1. Harp, J.B., et al. 2001. Differential expression of signal transducers and activators of transcription during human adipogenesis. Biochem. Biophys. Res. Commun. 281: 907-912. 2. Zhang, Y., et al. 2015. Down-regulated long non-coding RNA MEG3 and its effect on promoting apoptosis and suppressing migration of trophoblast cells. J. Cell. Biochem. 116: 542-550. 3. Lv, O., et al. 2016. Mild hypothermia protects against early brain injury in rats following subarachnoid hemorrhage via the TrkB/ERK/CREB signaling pathway. Mol. Med. Rep. 14: 3901-3907. 4. Dehghan, E., et al. 2017. Hydralazine induces stress resistance and extends. C. elegans lifespan by activating the NRF2/SKN-1 signalling pathway. Nat. Commun. 8: 2223.. 5. Weng, H., et al. 2018. METTL14 inhibits hematopoietic stem/progenitor differentiation and promotes leukemogenesis via mRNA m6A modification. Cell Stem Cell 22: 191-205.. 6. Avagliano Trezza, R., et al. 2019. Loss of nuclear UBE3A causes electro-physiological and behavioral deficits in mice and is associated with Angelman syndrome. Nat. Neurosci. 22: 1235-1247. 7. De Pace, R., et al. 2020. Synaptic vesicle precursors and lysosomes are transported by different mechanisms in the axon of mammalian neurons. Cell Rep. 31: 107775. 8. Ge, N., et al. 2021. Upregulation of KCNMA1 facilitates the reversal effect of verapamil on the chemoresistance to cisplatin of esophageal squamous cell carcinoma cells. Eur. Rev. Med. Pharmacol. Sci. 25: 1869-1880.

anti-plakoglobin mouse monoclonal (Progen, Heidelberg, Germany): validation for following manufacturers homepage: Application: ICC/IF, IHC, WB; Reactivity: Bovine, Dog, Human, Mouse, Rat, Zebrafish; validated for WB in human keratinocytes in "Role of Dsg1- and Dsg3-Mediated Signaling in Pemphigus Autoantibody-Induced Loss of Keratinocyte Cohesion", Walter et al, 2019, Front Immunol.

anti-desmoplakin rabbit polyclonal (Abclonal, MA, USA): recommended application: WB, IHC, species reactivities: Human, Mouse, Rat. Immunogen against: Recombinant fusion protein containing a sequence corresponding to amino acids 370-545 of human Desmoplakin (NP_004406.2). Validated for WB und IHC. References provided by reviewers: Pemphigus Foliaceus Autoantibodies Induce Redistribution Primarily of Extradesmosomal Desmoglein 1 in the Cell Membrane, Hiermaier et al, Front Immunol, 2022.

anti-α-Tubulin mouse monoclonal (Abcam, Berlin, Germany): species reactivitiy: mouse, rat, human; tested applications: Flow Cyt, ICC/IF, IHC-P, WB, 887 references provided by manufacturer, e.g Brunetta, Henver S et al. "Nitrate consumption preserves HFD-induced skeletal muscle mitochondrial ADP sensitivity and lysine acetylation: A potential role for SIRT1." Redox biology vol. 52 (2022): 102307. doi:10.1016/j.redox.2022.102307

anti-p-plakoglobin S665 mouse monoclonal: antibody was validated using plakoglobin KO murine cardiomyocytes and keratinocytes, please refer to: Yeruva, S. et al. Adrenergic Signaling-Induced Ultrastructural Strengthening of Intercalated Discs via Plakoglobin Is Crucial for Positive Adhesiotropy in Murine Cardiomyocytes. Front Physiol 11, 430, doi:10.3389/fphys.2020.00430 (2020).

anti-desmoglein-3 mouse monoclonal Invitrogen, Carlsbad, CA, USA): Specificity was tested in our laboratory in human keratinocytes deficient for Dsg3 (Walter, E. et al., Front Immunol, 2019). Relevant citation: Mao, Xuming et al. "p38 MAPK activation is downstream of the loss of intercellular adhesion in pemphigus vulgaris." The Journal of biological chemistry vol. 286,2 (2011): 1283-91. doi:10.1074/jbc.M110.172874

anti-occludin rabbit polyclonal (Invitrogen, Carlsbad, CA, USA), Reactivity: dog, human, rat, mouse; Tested Applications: WB, IHC, IF; Relevant Citation: Zhou, Ming-Yue et al. "Diprotin A TFA Exerts Neurovascular Protection in Ischemic Cerebral Stroke." Frontiers in neuroscience vol. 16 861059. 9 May. 2022, doi:10.3389/fnins.2022.861059

anti-desmoglein-1 rabbit polyclonal (Abclonal, MA, USA), validation data for WB and IF; Reactivity: Human, Rat, Tested applications: WB, IF

anti-Desmoglein-1 mouse monoclonal (Progen, Heidelberg, Germany), recommended applications: Immunohistochemistry (IHC) - frozen Undiluted-1:50 (preincubation (directly after fixation) with 0.05-0.2%, Triton X-100 for 5-10 min, depending on tissue type, recommended), Immunohistochemistry (IHC) - paraffin Undiluted-1:50 (preincubation with 0.001% trypsin and addition of 2%, milk powder to the incubation media and microwave treatment recommended), Western Blot (WB) Assay dependent

anti-E-cadherin mouse monoclonal (BD, Eysins, Switzerland), Reactivity: Human (QC Testing), Mouse, Rat, Dog (Tested in Development), Application: Western blot (Routinely Tested), Immunofluorescence, Immunohistochemistry, Immunoprecipitation (Tested During Development);

anti-β-catenin mouse monoclonal (BD, Eysins, Switzerland), QC Testing: Human, Tested in Development: Mouse, Rat, Dog, Chicken; Application: WB (routinely tested), IP, IF,IHC (tested during development), five references provided by manufacturer e.g Eger, A et al. "Epithelial mesenchymal transition by c-Fos estrogen receptor activation involves nuclear translocation of beta-catenin and upregulation of beta-catenin/lymphoid enhancer binding factor-1 transcriptional activity." The Journal of cell biology vol. 148,1 (2000): 173-88.

anti-panCK- FITC mouse monoclonal (Sigma Aldrich, St. Louis, MO), species reactivity: human, mouse, rat, bovine, frog, kangaroo rat, technique(s): direct immunofluorescence: 1:50 using PtK2 cells, immunohistochemistry (formalin-fixed, paraffin-embedded sections): 1:50 using human placenta, western blot: suitable.

anti-panCK mouse monoclonal (Sigma Aldrich, St. Louis, MO), species reactivity: human, mouse, rat, bovine, frog, kangaroo rat, technique(s): immunohistochemistry (formalin-fixed, paraffin-embedded sections): suitable using protease-digested sections of human or animal tissues, immunohistochemistry (frozen sections): suitable, indirect immunofluorescence: 1:400 using protease-digested, formalin-fixed, paraffin-embedded sections of human or animal tissues, western blot: suitable.

anti-CK14 mouse monoclonal (Abcam, Berlin, Germany), This antibody labels the basal layer of stratifying squamous and non-squamous epithelia. The staining pattern iscytoplasmic. It recognizes basal cell carcinomas and squamous cell carcinomas. Suitable for: WB, IHC-P, ICC, Reacts with: Human. Relevant Citation: Yang, Siqin et al. "Cell dynamics in Hertwig's epithelial root sheath are

regulated by β-catenin activity during tooth root development.” Journal of cellular physiology vol. 236,7 (2021): 5387-5398. doi:10.1002/jcp.30243
anti-Loricrin rabbit polyclonal (Abcam, Berlin, Germany): Validated for ICC, IHC-P, Species reactivity: Mouse, Human. 28 references provided by manufacturers e.g Zhou, Qian et al. “Phenformin Promotes Keratinocyte Differentiation via the Calcineurin/NFAT Pathway.” The Journal of investigative dermatology vol. 141,1 (2021): 152-163. doi:10.1016/j.jid.2020.05.114
anti-Dsc1 rabbit polyclonal (Abbexa, Cambridge, UK): tested applications: WB, IHC, IF/ICC, reactivity: human; validated in this study for IF in mouse.
anti-Dsc3 rabbit polyclonal (LSBio, Seattle, USA): reactivity: human, mouse; validated for IHC and WB

Eukaryotic cell lines

Policy information about cell lines and Sex and Gender in Research

Cell line source(s)	HaCaT cells were derived from Boukamp lab (Boukamp P, Petrussevska RT, Breitkreutz D, Hornung J, Markham A, Fusenig NE. Normal keratinization in a spontaneously immortalized aneuploid human keratinocyte cell line. J Cell Biol. (1988) 106:761–71. 10.1083/jcb.106.3.761) ,HaCaT cells stably transfected with keratin5-YFP (kind gift of Reinhard Windoffer and Nicole Schwarz, Institute of Molecular and Cellular Anatomy, RWTH Aachen University), murine keratinocytes were established in our laboratory. Analysis of Sex was not applicable due to age of mice used. NHEK were generated at Universitäts-Hautklinik Tübingen with written and informed consent of the donors. The procedure was approved by the medical ethical committee of the Eberhard Karls University Tübingen (ethical approval: EK318-21.) Keratinocytes of male and female donors behave similar in terms of epidermal blistering when treated with pemphigus vulgaris autoantibodies. Thus, we did not perform sex- and gender-based analysis of the keratinocytes.
Authentication	All cell lines were not authenticated.
Mycoplasma contamination	murine keratinocytes: mycoplasma free; human HaCaT: mycoplasma free; human HaCaT (keratin 5): mycoplasma positiv as tested by PCR
Commonly misidentified lines (See ICLAC register)	no commonly misidentified cell lines were used.

Animals and other research organisms

Policy information about studies involving animals; ARRIVE guidelines recommended for reporting animal research, and Sex and Gender in Research

Laboratory animals	mouse, BALB/cAnNCrl (in vivo experiments) , 2 days old; C57B1/6N (Knock-in mouse model for Jup S665A), 2 days old
Wild animals	The study did not involve wild animals.
Reporting on sex	For animal experiments not applicable due to age of mice used.<br>For ex vivo experiments: pemphigus show no prevalence to a certain sex/ gender and autoantibodies reveal comparable effects in both sexes. Furthermore, no morphological and compositional differences in the epidermis were observed between male and female samples. Thus, no gender specific data were obtained.
Field-collected samples	The study did not involve samples collected from the field.
Ethics oversight	Animal experiments and respective protocols were approved by an ethical board of Regierung von Oberbayern (Vet 02_21_205).

Note that full information on the approval of the study protocol must also be provided in the manuscript.
